# The biomarkers’ landscape of post-COVID-19 patients can suggest selective clinical interventions

**DOI:** 10.1038/s41598-023-49601-4

**Published:** 2023-12-15

**Authors:** Debora Paris, Letizia Palomba, Maria Cristina Albertini, Annabella Tramice, Lorenzo Motta, Eleonora Giammattei, Pasquale Ambrosino, Mauro Maniscalco, Andrea Motta

**Affiliations:** 1 Institute of Biomolecular Chemistry, National Research Council, 80078 Pozzuoli (Naples), Italy; 2 Department of Biomolecular Sciences, “Carlo Bo” University, 61029 Urbino, Italy; 3 Neuroradiology Unit, Ospedale Santa Maria Della Misericordia, 45100 Rovigo, Italy; 4 Directorate of Telese Terme Institute, Istituti Clinici Scientifici Maugeri IRCCS, 82037 Telese Terme (Benevento), Italy; 5 Pulmonary Rehabilitation Unit of the Telese Terme Institute, Istituti Clinici Scientifici Maugeri IRCCS, 82037 Telese Terme (Benevento), Italy; 6 Department of Clinical Medicine and Surgery, Section of Respiratory Disease, University of Naples Federico II, 80131 Naples, Italy; 7 Present Address: IRCCS Istituto Delle Scienze Neurologiche (Padiglione G), via Altura 3, 40139 Bologna, Italy

**Keywords:** Diagnostic markers, Predictive markers, Prognostic markers, Biomarkers, Molecular medicine

## Abstract

In COVID-19 clinical symptoms can persist even after negativization also in individuals who have had mild or moderate disease. We here investigated the biomarkers that define the post-COVID-19 clinical state analyzing the exhaled breath condensate (EBC) of 38 post COVID-19 patients and 38 sex and age-matched healthy controls via nuclear magnetic resonance (NMR)-based metabolomics. Predicted gene-modulated microRNAs (miRNAs) related to COVID-19 were quantified from EBC of 10 patients and 10 controls. Finally, clinical parameters from all post-COVID-19 patients were correlated with metabolomic data. Post-COVID-19 patients and controls showed different metabolic phenotype (“metabotype”). From the metabolites, by using enrichment analysis we identified miRNAs that resulted up-regulated (hsa-miR146a-5p) and down-regulated (hsa-miR-126-3p and hsa-miR-223-3p) in post-COVID-19. Taken together, our multiomics data indicate that post-COVID-19 patients before rehabilitation are characterized by persistent inflammation, dysregulation of liver, endovascular thrombotic and pulmonary processes, and physical impairment, which should be the primary clinical targets to contrast the post-acute sequelae of COVID-19.

## Introduction

Nearly 772 million people have been infected by the severe acute respiratory syndrome coronavirus-2 (SARS-CoV-2) as of November 8, 2023, including ca. 7 million deaths (https://covid19.who.int/), with more than 13.5 billion vaccine doses administered (as of November 5, 2023). SARS-CoV-2 induces a condition known as coronavirus disease 2019 (COVID-19), characterized by a wide range of clinical presentations and possible life-threatening complications^1^.

According to the National Institute for Health and Care Excellence (NICE) guidelines, different time phases of COVID-19 might be identified: “acute COVID-19 (signs and symptoms of COVID-19 for up to 4 weeks); ongoing symptomatic COVID-19 infection (signs and symptoms of COVID-19 from 4 to 12 weeks); and post-COVID-19 syndrome (signs and symptoms that develop during or after an infection consistent with COVID-19, continue for more than 12 weeks, and are not explained by an alternative diagnosis)”^2^. Overall, it is now clear that the convalescent phase of COVID-19 can present a number of clinical manifestations^3^ even in individuals who have had mild or moderate disease^4,5^.

Patients after COVID-19 may develop to so-called Long COVID, also referred to as “post-acute sequelae of COVID-19” (PASC)^6^. At least 70 million people around the world present long COVID^7–9^. They experience several symptoms, including cardiovascular, thrombotic and cerebrovascular disease, limited lung function with reduced lung capacities and volumes, respiratory muscle weakness, changes in radiographic and tomographic findings, type 2 diabetes, chronic fatigue syndrome, limitation in exercising, decreased functional capacity, and an overall reduced quality of life^9,10^. Symptoms can last for years^11^, with increasing public health costs and increasing economical burdening^12,13^.

For the pathogenesis of long COVID, several hypotheses have been put forward, including the persisting presence of SARS-CoV-2 in tissues, immune dysregulation with or without reactivation of underlying pathogens, alteration of the microbiota, microvascular blood clotting with endothelial dysfunction, among others^14^. The heterogeneity and complexity of post COVID-19 should be dealt with by specifically defining the targets for clinical interventions, with the aim of defining a multidisciplinary model of care to avoid burdening the patients and the health care systems with useless and costly over-investigation^15,16^. Physiological parameters obtained from a multiomics strategy can carefully define the patients’ status during the COVID-19 phases, recognizing and defining biological features related to and most likely predicting long-COVID manifestations^14^.

In this paper, we investigated the biomarkers’ landscape of long-COVID patients with several omics approaches to uncover molecular parameters that could suggest specific clinical management. We first defined the phenotype difference between patients and healthy controls by using nuclear magnetic resonance (NMR)-based metabolomics of their exhaled breath condensate (EBC)^17^ before entering a pulmonary rehabilitation (PR) that has been shown to be highly effective in improving the post-acute symptoms^18^. Such difference was also highlighted by assessing alterations in EBC-derived microRNAs (miRNAs) related to COVID-19. Finally, joining metabolomics data and clinical parameters collected during the rehabilitation program, we obtained a clear description of the pathophysiological condition of patients, highlighting the presence of persistent inflammation, dysregulation of liver, endovascular thrombotic and pulmonary processes, and physical impairment, which should be the primary targets in a management protocol of the post-acute sequelae of COVID-19.

## Results

### Patients

**Figure 1:**
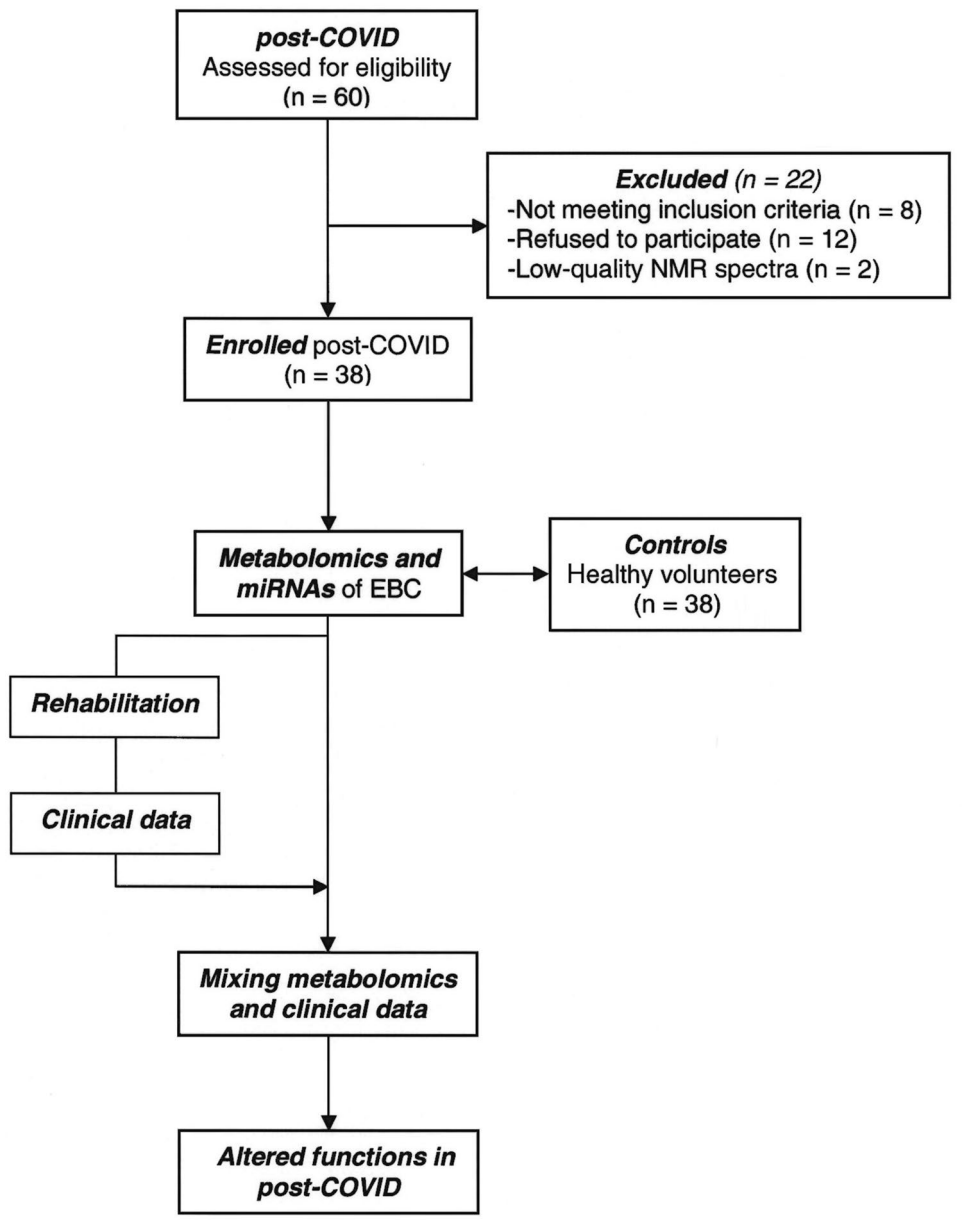
Schematic diagram illustrating the overall study design.

The study design is presented in Fig. 1. We screened 60 convalescent COVID-19 patients, all negativized from the wild-type SARS-CoV-2, which was the predominant form in South Italy at the time, although the presence of the D614G variant was also reported, but only 40 (92% males, mean age 58.8 years) were enrolled. Two out of 40 patients were excluded because of the low-quality NMR spectra. Samples and clinical data of 38 age- and sex-matched non-COVID-19 subjects (92% males, mean age 57.9 years) were also used as controls. They belonged to an irreversible deidentified Maugeri historical cohort of healthy volunteers selected from the hospital staff, whose samples (including EBC) and clinical data were previously collected and stored. The absence of significant respiratory, cardiac and/or metabolic diseases were anamnestic.

Their major demographic and clinical characteristics are reported in Table 1 as mean ± standard deviation (SD). All patients presented a long-COVID condition, with lingering, recurrent symptoms after recovering from the severe/critical condition. EBC samples and all clinical and instrumental data were collected from the 38 post-COVID patients before entering the rehabilitation cycle, and, in parallel, from the 38 control subjects. For the 38 patients, clinical and instrumental data were also collected after the rehabilitation cycle.

**Table 1:** Characteristics and clinical parameters of the subjects enrolled in the study.

Clinical data (units)		IN	OUT	Healthy subjects	p_IN-OUT_ value^a^
N	38	–	–	38	–
Sex (F/M)	3/35			3/35	–
Age (years)	58.82 ± 10.08	–	–	57.93 ± 11.23	–
Rehabilitation period (days)	24.32 ± 10.85	–	–	–	–
Hospitalization for acute cases (days)	14.30 ± 14.52	–	–	–	–
WHO, severe/critical (n)	17/21	–	–	–	–
Albumin (g/dL)		3.62 ± 0.50	3.71 ± 0.32	3.57 ± 0.47	ns
BMI (kg/m^2^)		29.74 ± 6.47	29.26 ± 5.47	25.33 ± 3.51	ns
Weight (kg)		90.02 ± 20.99	90.12 ± 21.59	85.68 ± 15.36	ns
Systolic pressure (mmHg)		127.57 ± 15.71	125.38 ± 9.48	120.42 ± 17.11	ns
Diastolic pressure (mmHg)		79.19 ± 9.82	75.00 ± 5.83	77.22 ± 11.01	0.031
TC (mg/dL)		191.90 ± 30.89	182.76 ± 33.21	186.61 ± 27.08	ns
TGs (mg/dL)		167.65 ± 70.72	165.76 ± 58.89	134.29 ± 16.04	ns
Glycemia (mg/dL)		95.86 ± 28.40	83.00 ± 17.08	81.19 ± 13.24	0.007
Creatinine (mg/dL)		0.85 ± 0.29	0.86 ± 0.28	0.79 ± 0.17	ns
Urea (mg/dL)		39.00 ± 14.73	33.85 ± 9.38	37.06 ± 6.23	0.009
Uricemia (mg/dL)		5.32 ± 1.82	5.51 ± 1.27	5.20 ± 0.56	ns
AST (U/L)		24.27 ± 14.62	18.42 ± 12.07	20.19 ± 5.81	1.90 × 10^–4^
ALT (U/L)		63.05 ± 68.13	43.61 ± 48.50	27.26 ± 12.32	8.83 × 10^–4^
CRP (mg/L)		15.05 ± 28.72	3.28 ± 4.78	2.56 ± 1.91	4.22 × 10^–6^
D-dimer (ng/mL)		727.78 ± 684.12	483.34 ± 405.31	319.36 ± 85.57	6.22 × 10^–4^
Red blood cells (10^12^/L)		4.52 ± 0.66	4.43 ± 0.90	4.81 ± 0.36	ns
Hemoglobin (g/dL)		12.78 ± 1.77	12.34 ± 1.97	12.27 ± 2.22	ns
Hematocrit (%)		38.98 ± 4.95	38.07 ± 5.73	37.70 ± 4.29	ns
Platelets (10^9^/L)		203.40 ± 77.41	190.86 ± 50.89	224.06 ± 24.31	ns
Leucocytes (10^9^/L)		8.13 ± 3.24	7.91 ± 3.84	5.70 ± 1.61	ns
PaO_2_ (mmHg)		72.52 ± 13.50	81.11 ± 11.53	–	1.12 × 10^–4^
PaCO_2_ (mmHg)		35.76 ± 3.14	36.67 ± 2.50	–	ns
pH		7.44 ± 0.02	7.44 ± 0.05	–	ns
HCO_3_ (mEq/L)		25.02 ± 1.37	25.87 ± 3.60	–	ns
SpO_2_ (%)		93.95 ± 3.45	96.00 ± 2.03	97.03 ± 1.05	2.53 × 10^–4^
FEV_1_ (L)		2.29 ± 0.67	2.62 ± 0.66	–	8.86 × 10^–6^
FEV_1_ (% predicted)		73.11 ± 19.37	82.12 ± 16.88	–	3.85 × 10^–5^
FVC (L)		2.78 ± 0.82	3.24 ± 0.77	–	1.86 × 10^–5^
FVC (% predicted)		70.73 ± 18.92	81.59 ± 15.64	–	2.90 × 10^–5^
FEV_1_/FVC		82.94 ± 7.09	81.12 ± 6.61	–	0.0026
DLCO (mL/min/mmHg)		16.27 ± 6.02	18.77 ± 6.96	–	0.001
DLCO/VA (mL/min/mmHg)		3.68 ± 0.76	3.90 ± 0.68	–	9.91 × 10^–4^
6MWD (m)		200.87 ± 133.34	346.11 ± 119.85	526.25 ± 87.89	7.28 × 10^–11^
CAT		26.45 ± 3.52	8.68 ± 4.04	–	7.28 × 10^–12^
Barthel index		77.71 ± 22.31	96.82 ± 7.48	–	2.33 × 10^–10^

IN and OUT refers to post-COVID patients before (IN) and after (OUT) the rehabilitation program.

*WHO* World Health Organization class of severity, *BMI* body-mass index, *TC* total cholesterol, *TGs* triglycerides, *AST* aspartate aminotransferase, *ALT* alanine aminotransferase, *CRP* C-reactive protein, *HCO*_*3*_ actual bicarbonate, *PaO*_*2*_ partial pressure of oxygen in arterial blood, *PaCO*_*2*_ partial pressure of carbon dioxide, *FEV*_*1*_ forced expiratory volume during the first second of a forced breath, *FVC* forced vital capacity, *FEV*_*1*_*/FVC* ratio between the forced expiratory volume in the first second (FEV_1_) and the forced vital capacity (FVC) of the lungs, *DLCO* carbon monoxide diffusing capacity of the lung, *6MWD* six-minute walking distance, *CAT* chronic obstructive pulmonary disease (COPD) assessment test questionnaire, *Barthel index* scale used to measure performance in activities of daily living. The values are reported as mean ± SD.

^a^IN/OUT clinical parameters tested with paired Wilcoxon signed ranks test (*ns* not significant).

In brief, convalescent COVID-19 patients were middle-aged male subjects with a recent history of severe (44.7%) or critical (55.3%) COVID-19 according to World Health Organization (WHO) criteria (https://www.covid19treatmentguidelines.nih.gov/overview/clinical-spectrum). 63.2% of patients was transferred from an acute care setting after a hospitalization of 14.3 (7–47) days, while all 38 enrolled patients underwent a rehabilitation program of 24.3 (5–57) days (Table 1). Rehabilitation affected several clinical characteristics of post-COVID patients (*p*-value column in Table 1). Statistically significant variations were observed for the pulmonary parameters (PaO_2_, and from SpO_2_ down to Barthel index in Table 1), and for BMI, weight, diastolic pressure, glycemia, urea, uricemia, AST, ALT, CRP and D-dimer values, demonstrating the successful impact of rehabilitation.

### NMR-based metabolomics of patients’ EBC

**Figure 2:**
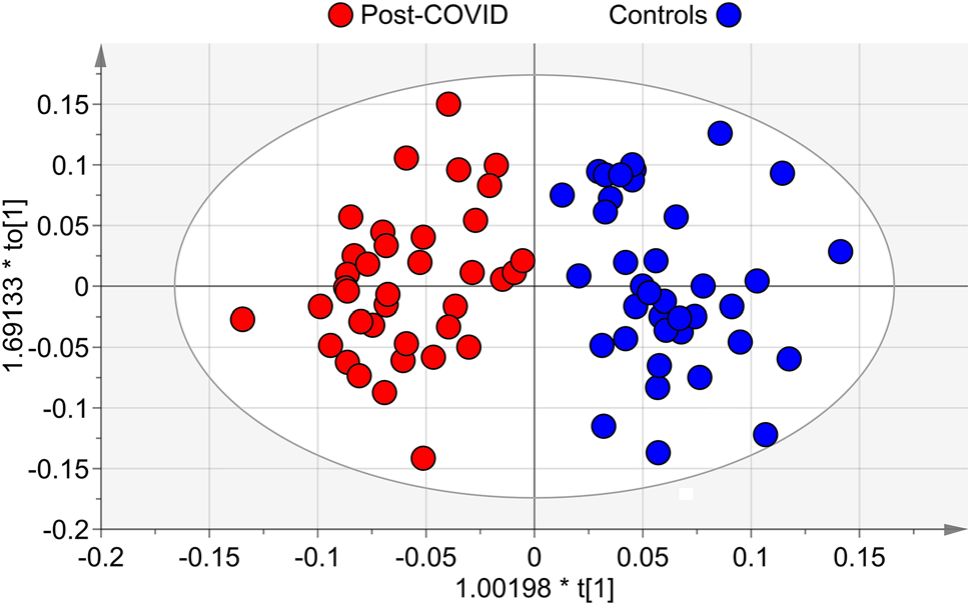
Orthogonal projections to latent structures discriminant analysis (OPLS-DA) of EBC samples from post-COVID patients and controls. Scores plot showing the degree of separation of the model between post-COVID (red circles) and controls (blue circles). The model presents strong regression (95%, CV-ANOVA *p* < 2.3 × 10^−12^) and high-quality parameters (R^2^ = 81% and Q^2^ = 87%). The labels t[1] and t_o_[1] along the axes represent the scores (the first 2 partial least-squares components) of the model, which are sufficient to build a satisfactory classification model.

To define the post-COVID physiological state, we profiled by NMR the EBC from patients, and compared them with the corresponding profiles of healthy subjects. Figure S1 compares the NMR spectra of the EBC samples from a healthy subject (a) with that of a patient (b), and resonances’ assignments are reported in Table S1. Notably, saliva contamination was absent in both samples as the most intense saliva signals, originating from carbohydrates and resonating between 3.3 and 6.0 ppm, are absent. PCA was used to explore data trend and possible outliers (data not shown). We then carried out supervised OPLS-DA, which yielded a regression model with high-quality parameters (R^2^ = 0.81, Q^2^ = 0.87 and CV ANOVA *p* = 2.3 × 10^−12^), and a clear class discrimination (Fig. 2). In the associated loadings plot (not shown), the post-COVID group, with respect to controls, presented upregulation of ethanol, lactate and acetoin, and downregulation of acetate, acetone, fatty acids, isocaproate, isovalerate, methanol and valerate. Their statistical significance is reported as box and whiskers plots in Supplementary Figs. 1–3. These results indicate that patients present a metabotype completely different from that of healthy subjects.

The discriminating biomarkers were used to identify the metabolic networks altered in post-COVID. Application of enrichment metabolic analysis indicated the potential biological mechanisms producing the separation between post-COVID and controls. With a threshold of *p* < 0.05, we uncovered synthesis and degradation of ketone bodies, pyruvate metabolism, propanoate metabolism, butanoate metabolism, cAMP signaling pathway, inflammatory mediator regulation of TRP channels and carbon metabolism as the most probable activated pathways. They mark the differences between the post-COVID-19 metabotype with respect to controls. The results of the enrichment analysis are reported as Supplementary Table S2.

### miRNA analysis

Potential genes related to altered metabolites found in EBC were derived from gene-metabolite interaction network analysis (Supplementary Table S3). Putative miRNAs involved in the modulation of the found genes were uncovered by an in silico analysis using the miRNet tool. This approach integrated the metabolomic analysis and miRNAs modulation in the same samples. Enrichment analysis based on the hypergeometric test explored 20 miRNA functions significantly modulated (*p* < 0.05). Validation of miRNAs through qRT-PCR was obtained considering the functions cell cycle (74 hits, Gene ontology (GO) annotations number GO:0007049), regulation of stem cell proliferation (74 hits, GO:0072091), cell death (73 hits, GO:0008219), aging (70 hits, GO:0007568), hematopoiesis (68 hits, GO:0030097) and angiogenesis (66 hits, GO:0001525) (Supplementary Table S4).

**Figure 3:**
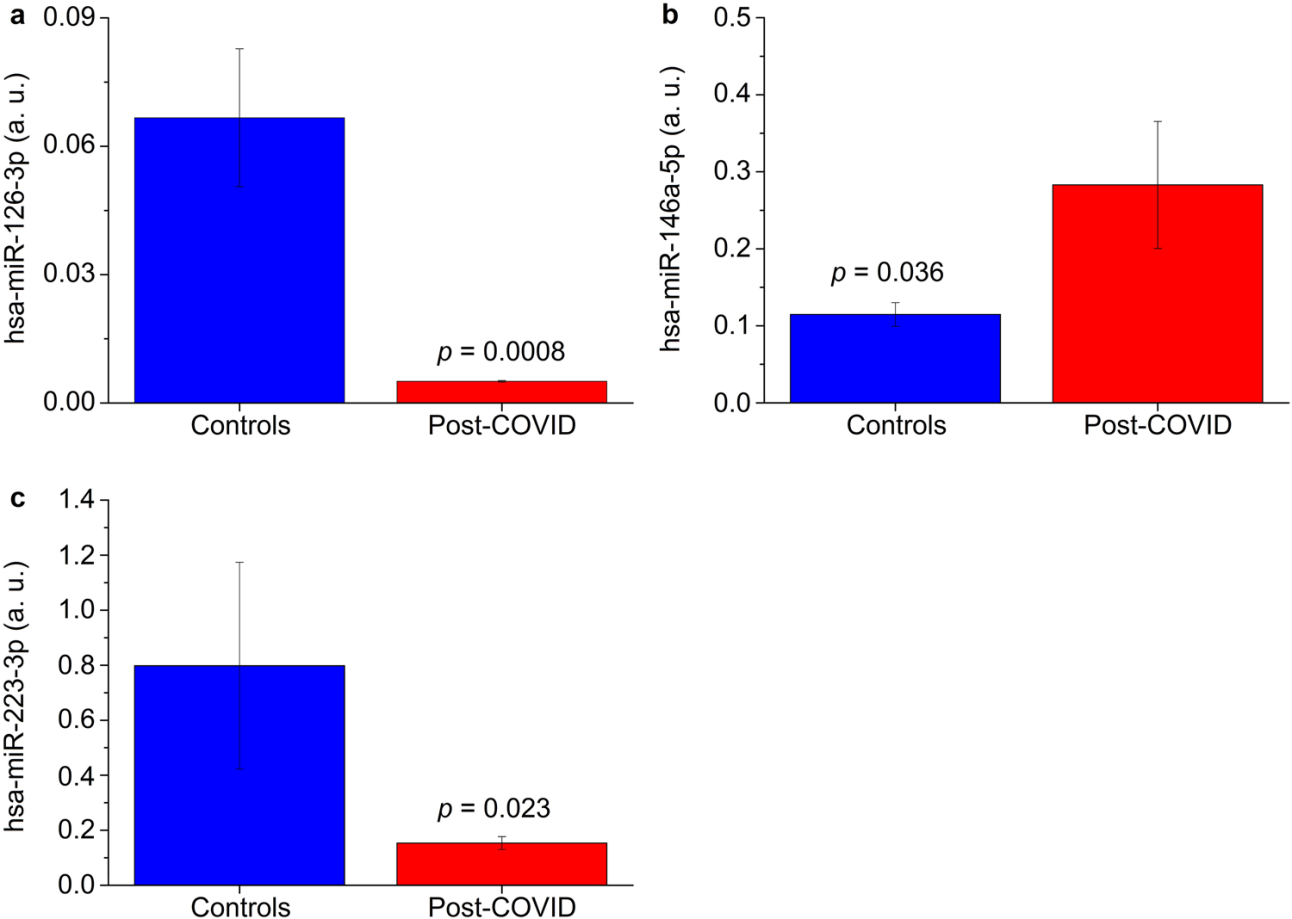
Relative expression of miRNAs in EBC samples obtained from enrolled subjects. (**a**) hsa-miR-126-3p-3p. (**b**) hsa-miR-146a-5p-5p. (**c**) hsa-miR-223-3p-3p. Blue bars refer to control subjects, while red bars refer to post-COVID patients. Analysis was performed with qRT-PCR. *p*-values are shown.

Among the miRNAs associated with the above functions, we identified hsa-miR-145-5p, hsa-miR-221-3p, hsa-miR-221-5p, hsa-miR-17-5p, hsa-miR-222-3p and hsa-miR-34a-5p common to all six functions, hsa-miR-146a-5p common to five functions, and hsa-miR-126-3p and hsa-miR-223-3p common to four functions. A PubMed search (“miRNAs name” AND “COVID-19”) indicated that hsa-miR-34a-5p, hsa-miR-146a-5p, hsa-miR-126-3p and hsa-miR-223-3p are associated with COVID-19 pathogenesis, which we searched for in EBC samples of post-COVID-19 patients. Except for hsa-miR-34a-5p, which was below the limit of detection (Cq ≥ 35) in more than 80% of samples and therefore was not considered further, different modulation was found for the other three miRNAs. Compared with healthy controls, patients presented up-regulation of hsa-miR146a-5p (Fig. 3b), while hsa-miR-126-3p and hsa-miR-223-3p were down-regulated (Fig. 3a,c, respectively). They are involved in inflammatory responses and immune regulations, and their alterations in post-COVID-19 indicate the persistence of pathophysiological processes.

### Correlation of EBC metabolites with clinical parameters

**Figure 4:**
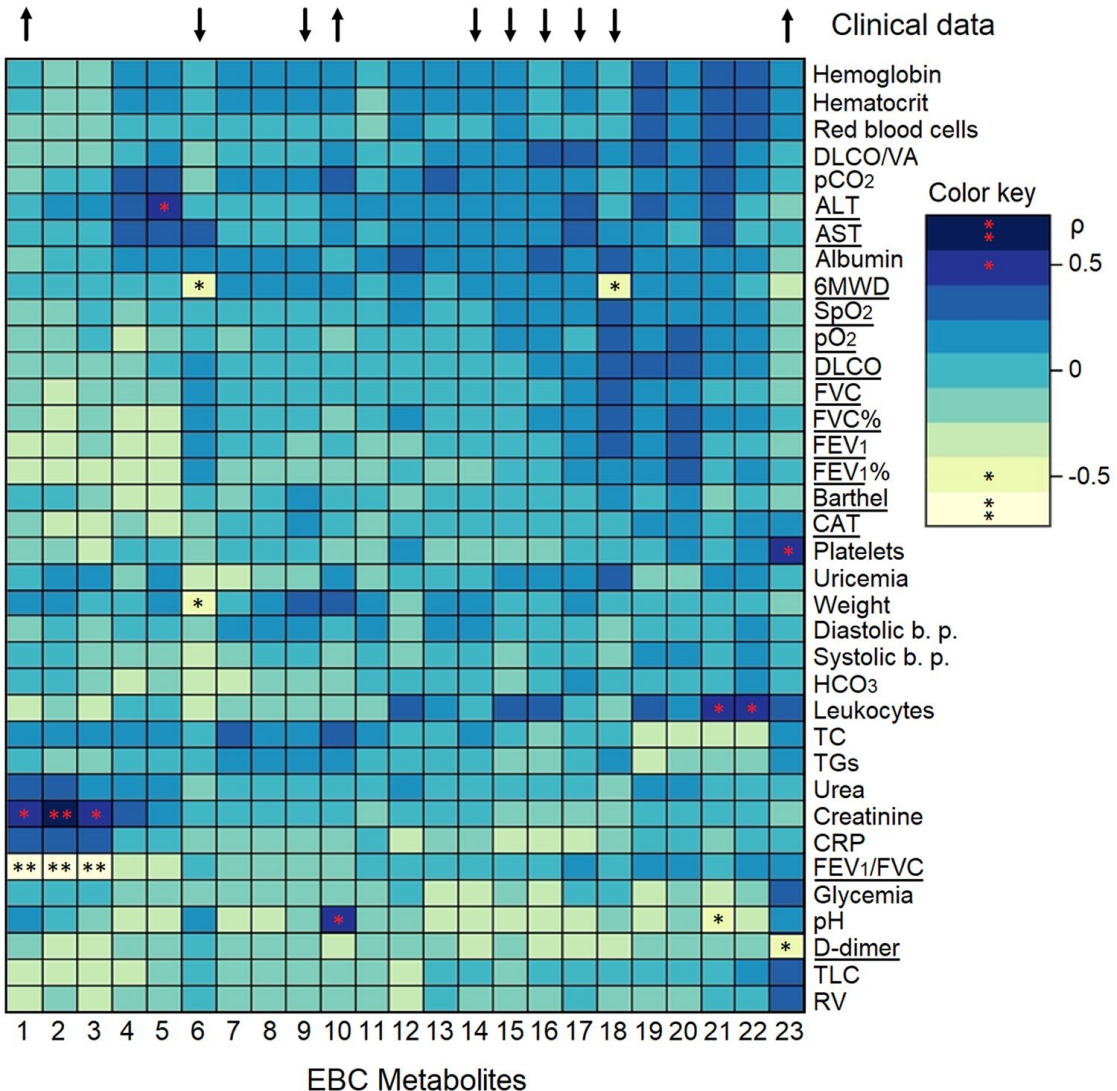
Heatmap based on Pearson correlation coefficients between EBC metabolites and values obtained from clinical test in negativized COVID-19 patients. Rows and columns are rearranged according to the centroid-based correlation matrix-based hierarchical clustering (CMBHC). Blue tone indicates positive correlations between metabolites and clinical data, whereas light tones indicate negative correlations. Correlation values ρ = |0.7| are marked with a double asterisk, while values ρ = |0.5| are labeled with a single asterisk. EBC metabolites are: 1, acetoin; 2, propionate/isobutyrate; 3, propionate/valine; 4, pyruvate; 5, isobutyrate; 6, methanol; 7, 3-hydroxyisovalerate; 8, isovalerate; 9, fatty acids (FA); 10, lactate; 11, formate; 12, trimethylamine; 13, 2-hydroxyisovalerate; 14, isocaproate; 15, isovalerate; 16, valerate; 17, acetate; 18, acetone; 19, serine; 20, isopropanol; 21, glycine; 22, 3-hydroxybutyrate; 23, ethanol. The arrows on top label significant EBC metabolites, whose trend, with respect to healthy subjects, is symbolized by the arrow direction. Statistically significant clinical data are underlined.

The post-COVID-19 metabolites from EBC were associated with the clinical parameters obtained at the hospitalization before rehabilitation. The heatmap in Fig. 4 shows the significant Pearson correlation coefficients (*p* < 0.05) between the metabolites and at least one clinical parameter. Considering a threshold value of ρ ≥ |0.5|, we identified a positive correlation of 0.7 between propionate/isobutyrate (label 2 in Fig. 4) and creatinine (dark blue box with a red double asterisk, see the color code in Fig. 4). Positive correlations of 0.5 were observed between acetoin (label 1) and propionate/valine (label 3) with creatinine, isobutyrate (label 5) and alanine aminotransferase (ALT), lactate (label 10) and pH, glycine (label 21) and leukocytes, 3-hydroxyisobutyrate (label 22) and leukocytes, and ethanol (label 23) with platelets (blue boxes with a red asterisk).

Negative correlations of − 0.7 were observed between acetoin, propionate/isobutyrate and propionate/valine with FEV_1_/FVC (labels 1, 2 and 3, respectively, pale yellow boxes with a black double asterisk). Negative correlations of − 0.5 involved methanol (label 6) with weight and the six-minute walking distance (6MWD), acetone (label 18) and 6MWD, and glycine (label 21) and pH (light green boxes with a black asterisk in Fig. 4). Positive correlation indicates similar behavior between metabolites and clinical values (increase/increase, decrease/decrease), while negative correlation refers to opposite behavior (increase/decrease, decrease/increase). Such correlations indicate that clinical parameters can be monitored via metabolites, which could become noninvasive markers of the clinical status.

### Rehabilitation of post-COVID-19 patients: analysis of the clinical data between admission and discharge

**Figure 5:**
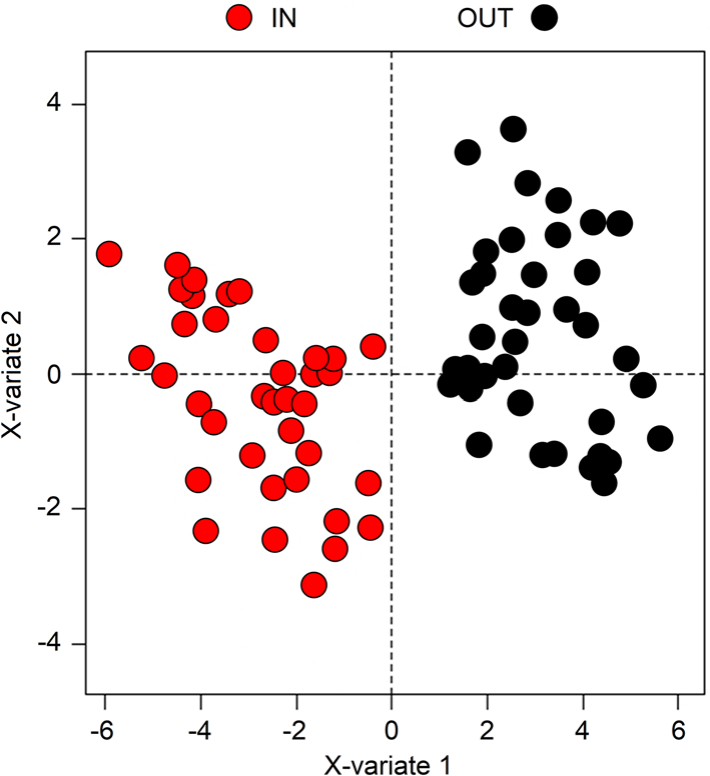
Multilevel PLS-DA scores plot for post-COVID patients. The labels X-variate 1 and X-variate 2 along the axes represent the scores (the first 2 partial least-squares components) of the model, which are sufficient to build a satisfactory classification model. Admission variables (IN) are shown in red, while discharge variables (OUT) are in black.

**Figure 6:**
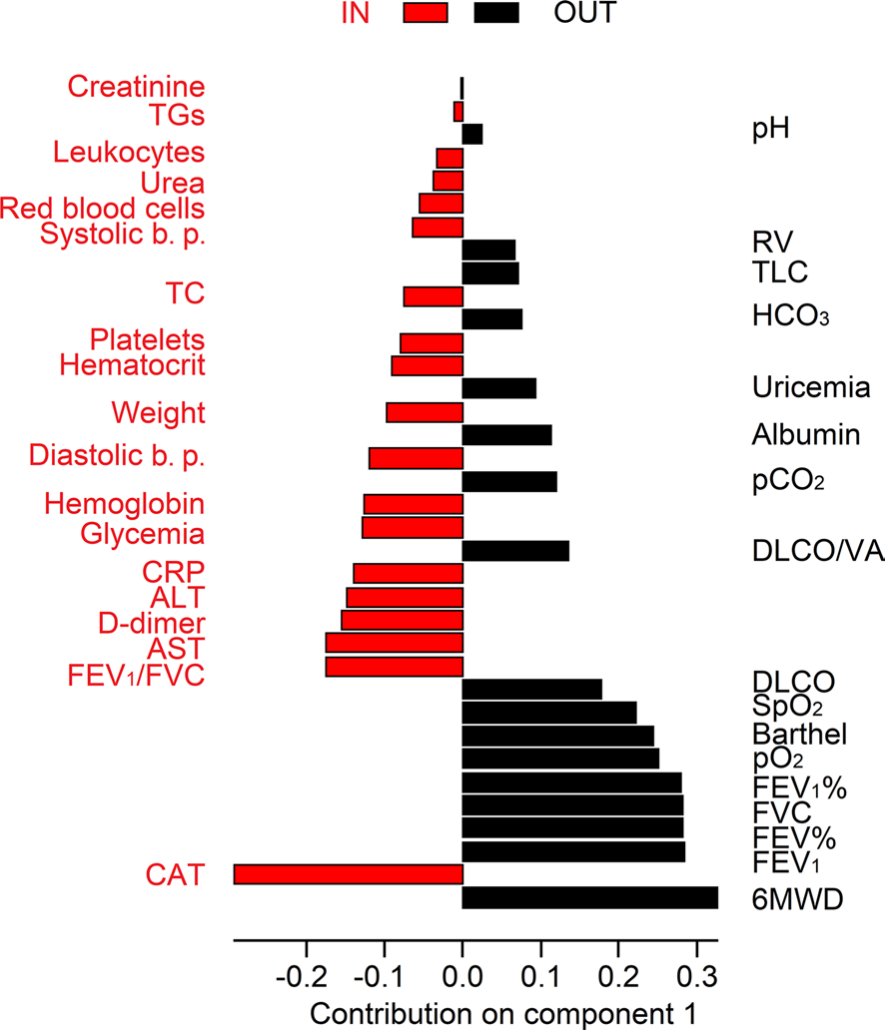
Contribution plot of the principal component PC1 of the multilevel PLS-DA model including the clinical parameters of the post-COVID patients. Each bar represents the loading value for each variable on PC1. Admission variables (IN) are shown in red, while discharge variables (OUT) are in black.

The effects of rehabilitation on patients were evaluated by comparing the clinical/laboratory data of each patient at the admission in an average rehabilitation cycle of 24.3 days (*in*) (Table 1) and at discharge (*out*). The scores multilevel PLS-DA plot of Fig. 5 shows that the discharge status (black dots, *out*) is different from the one at the admission (red dots, *in*). In particular, at the admission, patients presented higher values of creatine, triglycerides (TGs), leukocytes, urea, red blood cell count, systolic blood pressure, total cholesterol (TC), platelets, hematocrit, weight, diastolic blood pressure, hemoglobin, glycemia, C-reactive protein (CRP), ALT, D-dimer, aspartate aminotransferase (AST), FEV_1_/FVC and CAT. At discharge, patients were characterized by higher values of pH, total lung capacity (TLC), HCO_3_, uricemia, albumin, PaCO_2_, DLCO/VA, DLCO, SpO_2_, Barthel, PaO_2_, FEV_1_%, FVC, FVC%, FEV_1_ and 6MWD. This is depicted in Fig. 6, which reports the contribution plot related to the above multilevel PLS-DA model, where each bar represents the loadings value for each variable on the principal component PC1 at the admission (red bars) and at discharge (black bars).

Statistical significance was found for AST, ALT, D-dimer, CAT, Barthel, DLCO, SpO_2_, PaO_2_, FEV_1_/FVC, FEV_1_, FVC, FEV_1_%, FVC% and 6MWD (Table 1), which are the principal clinical parameters that are carried over upon negativization. Therefore, post-COVID-19 patients should be monitored for liver damage (AST and ALT), endovascular thrombotic processes (D-dimer), persisting pulmonary symptoms (CAT, Barthel, DLCO, SpO_2_, PaO_2_, FEV_1_, FVC, FEV_1_/FVC, FEV_1_%, FVC%), and physical impairment (6MWD). The relationship between the above parameters and the statistically significant EBC metabolites (top arrows in Fig. 4) indicated negative correlations between increased acetoin (label 1) and decreased FEV_1_/FEV (ρ =  − 0.7, underlined in Fig. 4), decreased methanol (label 6) and acetone (label 18) with increased 6MWD, increased ethanol (label 23) and D-dimer (all presenting ρ =  − 0.5).

Taken together, the metabolomic, the miRNAs and the clinical data point out that post-COVID patients still present dysregulation of the liver, endovascular and pulmonary parameters.

## Discussion

Our results show that post-COVID-19 patients present several dysfunctions from which post-acute sequelae could originate. In particular, the post-COVID-19 group showed persistent lung inflammation as indicated by upregulation of ethanol, lactate and acetoin, and downregulation of acetate, methanol, acetone, fatty acids, isocaproate, isovalerate and valerate. In fact, increased acetoin level is associated with airway inflammation^19^, and reduction of methanol was observed in the EBC of lung cancer patients^20^. Short-chain fatty acids acetate, isovalerate, valerate and isocaproate (SCFAs) are involved in the regulation of several leukocyte functions linked to the production of cytokines, eicosanoids and chemokines, and are reported to affect leukocyte migration to the inflammation foci^21^. Acetone and lactate were detected in the bronchoalveolar lavage fluid of cystic fibrosis patients with varying levels of inflammation^22^. In addition, lactate excess can bring about a noticeable raise in ROS and apoptosis in A549 alveolar cells^23^. It was reported that non-survivor COVID-19 patients had higher lactate levels with respect to survivors at the intensive-care unit admission^24^. Furthermore, lactate is the main downgrading product of anaerobic metabolism, and it is well known that COVID-19 patients present hypoxic lung damage and respiratory failure, and that hypoxia is an indicator of COVID-19 mortality^25^. Significantly different concentrations between COVID-19 patients within 21 days from clinical diagnosis and post-COVID-19 groups were observed for acetate, acetone and lactate also in plasma^26^.

Correlation of EBC metabolites with clinical data from patients showed statistically significant relationships between increased acetoin and reduced FEV_1_/FVC (ρ =  − 0.7), decreased methanol and acetone with increased 6MWD (ρ =  − 0.5), and increased ethanol and decreased D-dimer (ρ =  − 0.5), which indicate that these metabolite alterations are manifestations of the corresponding physiological functions. As a confirmation, reduction of methanol and acetone and the corresponding 6MWD increase was observed in chronic obstructive pulmonary disease (COPD) patients after a 5-week rehabilitation program^27^, and ethanol can reduce the global fibrinolytic capacity of whole blood, measured as D-dimer production during incubation of blood clots^28^.

From the above metabolites we identified the most probable dysregulated metabolic pathways, namely synthesis and degradation of ketone bodies, pyruvate metabolism, propanoate metabolism, butanoate metabolism, cAMP signaling pathway, and inflammatory mediator regulation of TRP channels. Interestingly, upregulation of ketone bodies and pyruvate metabolisms has been observed in previous NMR-based metabolomics studies of serum/plasma samples from post-COVID patients^26,29–31^.

Ketone bodies (KBs) are produced by hepatocytes’ mitochondria where fatty acids enter upon adipocytokine signaling. Interestingly, two adipocytokines, IL-6 and tumor necrosis factor-alpha (TNFα), are related to COVID-19 severity and patients’ death^32^. Degradation of KBs (ketolysis) implies elevated levels of KBs in the blood and urine (ketosis). Ketosis shows an anti-inflammatory activity since β-hydroxybutyrate (β-HB), derived from the KB acetoacetate, is a key regulator of inflammation pathways like the NLRP3 inflammasome^33^. It has been suggested that in SARS-CoV-2 infection, treatments increasing β-HB levels could improve host defenses against respiratory viral infection while decreasing inflammation^34^. Additionally, the high levels of triglycerides and triglycerides-rich lipoproteins observed in COVID plasma^26^ could be generated by a limited oxidation of acetyl-CoA inside the mitochondria, therefore favoring the synthesis of ketone bodies and the high levels of β-HB, acetoacetate and acetone in COVID-19 patients^35^.

cAMP is involved in several inflammatory pathways, being able to inhibit ROS generation and proinflammatory cytokine production, primarily IL-6 and TNF-α^36^. Furthermore, preserving the cAMP concentration in the pulmonary tissue can improve lung functions^36^, which are essential in COVID-19 patients. Interestingly, anosmia and ageusia, which have been observed in COVID-19 patients, have also been related to the intracellular levels of cAMP^37^.

The propanoate and butanoate metabolisms describe the metabolism of the SCFAs propionate and butyrate. SCFAs mediate the communication between the intestinal microbiome and the immune cells via free fatty acid receptors (FFARs), and dysregulation of the FFAR2/3 receptors’ expression favored the insurgence of respiratory diseases^38^. We have observed that post-COVID 19 patients showed, with respect to controls, alteration of acetate, fatty acids, isocaproate, isovalerate, valerate (all SCFAs), and fatty acids, which are involved in the production of cytokines, eicosanoids, and chemokines responsible for the lung hyperinflammation in severe COVID-19 patients^39^.

Transient receptor potential (TRP) channels are widely expressed in tissues that are infected by SARS-CoV-2 and have been proposed as targets for adjuvant therapies against COVID-19^40^. Most of the clinical manifestations of COVID-19 activate different TRP channels. For example, TRPV4 is involved in the recruitment of neutrophils and macrophages during lung injury^41^ and relates to hearing loss/impairment^40^. Loss of either TRPM4 or TRPM5 channels may significantly impair taste^42^ and olfaction^43^. TRP channels also contribute to several cardiac complications (arrhythmias, cardiac fibrosis and myocyte hypertrophy) observed in COVID-19 patients^44^.

Using miRNet, from the discriminating metabolites we identified the perturbed genes, which in turn prompted the miRNAs altered in EBC. miRNAs have emerged as regulators of COVID-19^45,46^. In particular, we found hsa-miR-126-3p and hsa-miR-223-3p downregulated in post-COVID-19, while hsa-miR-146a-5p was upregulated. They are involved in the regulation of ACE2, the binding site of the virus, and in the inflammatory responses and immune regulation^47^. hsa-miR-126-3p attenuates lung inflammation via different pathways that reduce many proinflammatory cytokines including IL-6^48^, which in COVID-19 has been linked to high mortality risk^32^. In COVID-19 patients, the serum level of hsa-miR-126-3p was considerably reduced with the increase of disease grade^49^, and this pattern was also observed in patients non-responsive to therapies^50^. hsa-miR-126-3p downregulation was also detected in plasma samples of COVID-19 patients with respect to a healthy control group, while no downregulation was observed between severe and mild patients^51^, which was instead previously reported^52^. Furthermore, a positive correlation between miR-126-3p and neutrophils levels, and a significant negative correlation with IL-6 and D-dimer were observed^53^. Interestingly, in vitro hsa-miR-126-3p exhibited neutralizing activity against SARS-COV-2 infection^49^.

We here found that hsa-miR-126-3p does not return to the pre-COVID-19 values, and this is an indication of the persistent inflammation status after negativization. hsa-miR-126-3p also shows a pro-angiogenic role by stimulating endothelial cell proliferation^54^. The post-acute COVID-19 syndrome is associated with a persistent endothelial dysfunction, directly correlated with the severity of pulmonary impairment^55^, whose recovery is normally related to maintaining the physiological endothelial functions. Therefore, in line with the above results, the decrease we observed for hsa-miR-126-3p suggests the persistent presence of endothelial damage in patients.

Serum hsa-miR-223-3p directly inhibits the viral S protein expression and SARS-CoV-2 replication^56^, and is implicated in the regulation of inflammatory responses by inhibiting the action of the NLRP3 inflammasome and modulating the expression of inflammatory chemokines and cytokines^57^. In a possible mechanism, decrease of the has-miR-223-3p expression should increase NLRP3 expression levels and promote pyroptosis^58,59^. Furthermore, serum miR-223-3p Therefore, the reduced level of hsa-miR-223-3p observed in post-COVID-19 patients confirms that inflammation is still present after negativization. Interestingly, hsa-miR-223-3p was amplified by long-term physical exercise^56^, and we here found that 6MWD is the most important factor that characterizes the hospital discharge after post-COVID-19 rehabilitation. Taken together, this suggests a beneficial action of hsa-miR-223-3p with the consequent reduction of inflammation^56^.

Upon a viral infection, has-miR-146a is primarily produced to regulate the innate immune response and inflammation by negatively regulating the NF-κB pathway^60,61^. Therefore, its expression in COVID-19 decreases inflammatory disorders in target organs such as the lungs, heart, brain, skin, and underlying vascular disease^61,62^. The hsa-miR-146a-5p increase we observed in post-COVID-19 patients is an indication of the path to recovery, as the levels of IL-1, IL-6 and TNF-α cytokines are inversely correlated to has-miR-146a production^63,64^. In fact, hsa-miR-146a-5p was found *ca.* threefold higher in a COVID-19 post-acute group than in the acute group^65,66^, and COVID-19 patients who did not respond to tocilizumab treatment presented a reduction of has-miR-146a-5p with respect to responders, and its reduction in non-responders was associated to a higher risk of adverse outcomes^53^.

The above miRNAs are involved in cell cycle, regulation of stem cell proliferation, cell death, aging, hematopoiesis, and angiogenesis functions. Although nonspecific, cell cycle, regulation of stem cell proliferation and cell death could reflect the impact of COVID-19 on several multiorgan cellular processes, in line with the results of a proteomic analysis of autoptic samples from seven organs in COVID-19 patients^67^. Furthermore, the regulation of stem cell proliferation promotes remodeling and lung tissue regeneration after COVID-19-induced pneumonia and can help patients’ recovery^68^. Similarly, for cell death, acutely ill COVID-19 patients revealed an upregulation of cell death programs genes, acting in tissue specific manner^69^.

More specific are aging, hematopoiesis and angiogenesis. Aging is a main risk factor for severe COVID-19 and its worst outcomes because it induces immunosenescence, which hampers the response to the virus^70^, and inflammaging, a low-grade diffused inflammation^71^. Hematopoiesis alteration is associated with severe and fatal COVID-19 as SARS-CoV-2 alters the bone marrow microenvironment, weakening hematopoiesis and causing hemocytopenia^72^. Furthermore, IL-6, which increases dramatically in COVID-19, is important for regulation of hematopoiesis as it stimulates the production of bone marrow neutrophils^73^. Regarding angiogenesis, autoptic lungs from patients died from SARS-CoV-2 infection indicated the presence of significant new vessel growth and a corresponding differential upregulation of angiogenesis-associated genes^74^. Such a compensatory angiogenesis mechanism was also observed in heart, liver, kidney, brain and lymphoreticular organs in patients who died from COVID-19^75^.

Comparing clinical data from post-COVID-19 patients before and after the admission in a rehabilitation cycle, we detected dysregulation of parameters related to liver damage (AST and ALT), endovascular thrombotic processes (D-dimer), persisting pulmonary symptoms (CAT, Barthel, DLCO, SpO_2_, PaO_2_, FEV_1_, FVC, FEV_1_/FVC, FEV_1_%, FVC%), and physical impairment (6MWD). SARS-CoV-2-infected subjects present alterations of liver biochemistry^76^. Since AST and ALT increase is associated with the reduction of peripheral oxygen saturation in viral pneumonias^77^, it is expected that systemic hypoxia in COVID-19 may also alter AST and ALT levels. In fact, a five-fold increase of AST and ALT levels in COVID-19 with respect to normal is associated with an increased risk of death^78^, causing elevated levels of CRP (which is synthesized by the liver), D-dimer, ferritin and IL-6^76^. Therefore, the increased CRP values in patients before rehabilitation again confirms the persistence of liver alteration and inflammation.

Both venous and arterial thromboses characterize COVID-19 pathology^79^. D-dimer is an indirect marker of active coagulation and thrombin formation, and represents a mirror of the endovascular thrombotic processes. Higher levels of D-dimer are observed in severe patients infected with SARS-CoV-2 compared to nonsevere ones, and, significantly, increased D-dimer has been reported in COVID-19 nonsurvivors with respect to survivors, and the concentration continues to rise until death^80^.

The following limitations of the study should be considered. First, although the number of enrolled patients encompasses that indicated by backward analysis, our results depend on a relatively limited number of subjects (38 patients and 38 controls). For this reason, we combined different types of biomarkers (EBC, miRNAs and clinical parameters), which represent complementary physiological aspects. Second, the patients were not consecutively recruited since they were selected from those of the rehabilitation division. As such, parameters like hospitalization for acute cases and rehabilitation period were variable, ranging between 7 and 50 days, and 5 and 57 days, respectively. Furthermore, only 3 females (8%) were comprised in each group because the patients admitted were typically males. Also, the possibility that conditions/treatments not recorded because of the flexibility at the hospital admission affects our conclusions cannot be excluded. We are aware that such uncontrolled heterogeneity may contribute to variations in our findings, and therefore they need to be validated in a larger cohort of patients with more balanced parameters. However, such a heterogeneity reflected the emergency due to the COVID-19 pandemic being a real clinical setting. Third, although metabolomics was untargeted, miRNAs were assessed after comparing those derived from an in silico analysis with those related to COVID-19. Obviously, other miRNAs may have clinical significance, and their clinical role may have been underestimated. However, since individual miRNAs lack specificity and they should be used in combination with other omics parameters, we related miRNAs with metabolomics markers of EBC, which, to the best of our knowledge, has not been reported thus far.

Notwithstanding the above limitations, we were able to build a satisfactory description of the metabolic processes going on in post-COVID-19 patients, characterized by persistent inflammation, dysregulation of liver, endovascular thrombotic and pulmonary processes, and physical activities. A clear correlation was found between the metabolic response of patients and the clinical outcomes, which suggested selective interventions to face the pathophysiological status of patients and possibly contrast the post-acute sequelae of COVID-19. In addition, information on the rehabilitation could be obtained according to the biomarkers that characterize the post-COVID-19 metabotype.

All considered, the results shown here provide sufficient evidence that joining together breath metabolomics, miRNAs and clinical parameters can generate a reasonable understanding of the complex pathophysiological status of negativized SARS-CoV-2 patients. Our approach is basically noninvasive and could suggest an unbiased personalized approach to achieve an optimal use of healthcare resources.

## Methods

### Patients

Convalescent COVID-19 patients referring to the Pulmonary Rehabilitation Unit of Istituti Clinici Scientifici Maugeri IRCCS, Telese Terme, Italy, were screened from October 2020 to February 2021 for enrollment within 2 months of swab test negativization from the wild-type SARS-CoV-2, which was the predominant form in South Italy at the time, although the presence of the D614G variant was also reported. Inclusion criteria were: age ≥ 18 years; recent SARS-CoV-2 infection with severe-to-critical COVID-19 according to the NIH classification (https://www.covid19treatmentguidelines.nih.gov/overview/clinical-spectrum/); patients presenting a long-COVID condition, with lingering, recurrent symptoms after recovering from the severe/critical condition after a negative swab test; indication for a multidisciplinary rehabilitation program. Exclusion criteria were: recent (< 6 months) major surgery or any previous lung surgery; current malignancy; any history of chronic respiratory disease (e.g., asthma, COPD) other than COVID-19; inability to understand or sign the informed consent. Clinical data and EBC samples from age- and sex-matched healthy volunteers were also included in the study as controls. They belonged to an irreversible deidentified set of electronic Maugeri database containing records of people selected from the hospital staff, whose samples (including EBC) were previously collected and stored at − 80 °C. The absence of significant respiratory, cardiac and/or metabolic diseases were anamnestic.

Participants with missing data for the outcome of interest were excluded from the study.

We followed the STROBE reporting guidelines^81^, in line with the 1975 Declaration of Helsinki. The Ethic Committee of Istituto Nazionale Tumori, Fondazione Pascale, Naples, Italy approved the study (n. ICS 3/20).

### Study procedures

After signing the informed consent, all convalescent COVID-19 patients underwent a detailed collection of key demographic and clinical information related to the acute phase of COVID-19, lung function, physical performance, comorbidities and treatment(s). Following the same exclusion criteria as convalescent COVID-19 patients, data were extracted from an irreversibly de-identified electronic dataset for control subjects. Venous blood samples were used for the common hemato-chemical parameters. Arterial blood samples were collected to measure oxygen (PaO_2_) and carbon dioxide tension (PaCO_2_) using a blood gas analyzer (ABL 825® FLEX BGA, Radiometer Medical Aps, Copenhagen, Denmark). According to the protocols of the Spirometry parameters and diffusion lung capacity for carbon monoxide (DLCO) were also evaluated with an automated equipment (Vmax® Encore, Vyasis Healthcare, Milan, Italy) as reported^82,83^. Forced expiratory volume in 1 s (FEV_1_), forced vital capacity (FVC) and DLCO were expressed both as numerical values and percentages of predicted values (FEV_1_%, FVC% and DLCO%, respectively). The COPD Assessment Test (CAT)^84^ and the Barthel index were also administered to patients to evaluate the impact of the disease on daily living. Exercise capacity was tested by measuring the 6MWD^85^. All the clinical and the instrumental analyses were carried out at the admission (*in*) and at the discharge (*out*) after rehabilitation.

### Rehabilitation

The rehabilitation (a 5-week exercise-based program of 6 sessions/week (30 sessions)) protocol followed the official ATS/ERS guidelines (Supplementary Information)^86^. In brief, patients undertook a 5-week exercise-based program of 6 sessions/week (30 sessions). Physical exercise was the cornerstone of the program, which also included dietary and psychosocial counselling, based on treadmill walking, stationary cycling, arm ergometry, flexibility, stretching and strengthening exercises with body and fixed weights. The participation was monitored and supervised by a physiotherapist.

### EBC collection, NMR sample preparation and spectra acquisition

EBC samples were collected from negativized patients (post-COVID) before entering the rehabilitation program. Control samples were from a cohort of healthy volunteers belonging to an irreversible deidentified set of electronic Maugeri database containing records of people selected from the hospital staff, whose EBC samples were previously collected and stored at − 80 °C. The absence of significant respiratory, cardiac and/or metabolic diseases were anamnestic. Sample preparation and NMR spectra acquisition were carried out as described (Supplementary Information)^87,88^.

### Power analysis

In metabolomics, a priori power analysis is not feasible because concentration variations of biomarkers are unknown before analysis^89^. It was estimated by varying the 1 − α and 1 − β parameters from 95 to 99.9% and from 80 to 99.9%, respectively. Using the accuracy percentages obtained in our validation tests^89^, for 1 − α = 95% and 1 − β = 80% we derived 24 ± 3 post-COVID-19 patients for all classes, while for 1 − α = 1 − β = 99.9% we obtained 28 ± 3 patients. To account for possible drop-outs or protocol adherence problems, we screened 60 post-COVID-19 patients, with the final number of enrolled patients greater than those indicated by the backward analysis (40 *vs.* 24/28). However, 1 − α = 95% and 1 − β = 80%, and 99.9% is an extreme setting.

### Multivariate data analysis

EBC proton spectra were automatically subdivided into 420 discrete regions (‘buckets’) of equal width (0.02 ppm) and integrated (Supplementary Information)^87,88^. Each integral was normalized to the total spectrum area to account for possible dilution effects. NMR data were imported into SIMCA-P + 14 package (Umetrics, Umeå, Sweden) for Principal Components Analysis (PCA) and Orthogonal Projections to Latent Structures Discriminant Analysis (OPLS-DA) after Pareto scaling. Model quality was evaluated by using the goodness-of-fit parameter (R^2^) and the goodness-of-prediction parameter (Q^2^)^90^, together with an internal iterative 7-round cross-validation and permutation test (800 repeats) and ANalysis Of VAriance testing of Cross-Validated predictive residuals (CV-ANOVA). Quantification was achieved with OriginPro 9.1 software package (OriginLab Corporation, Northampton, USA). Statistical significance for selected metabolites was determined by parametric (ANOVA with Bonferroni correction) or non-parametric (Mann–Whitney U) tests according to the results of normality test performed to evaluate data distribution (Shapiro–Wilk, Kolgomorov–Smirnov test). *p* < 0.05 was considered statistically significant. To evaluate possible covariates, propensity score matching was used to further estimate discriminant metabolites between controls and post-COVID classes before rehabilitation. The propensity scores were estimated in R with the MatchIt package^91^ using logistic regression based on weight, systolic and diastolic pressure of Table 1, while considering the other variables for correlation purposes. One-to-one nearest neighbor matching was used and 37/38 patients were matched to a non-COVID subject in the dataset. Statistical differences in ethanol (*p* = 0.006), methanol (*p* = 0.003), acetone (*p* = 0.007), acetate (*p* = 2.05 × 10^−7^), acetoin (*p* = 0.01), lactate (*p* = 0.006), CH_2_ portion of fatty acids (*p* = 1.38 × 10^−7^), isovalerate (*p* = 1.02 × 10^−7^), valerate (*p* = 0.005) and isocaproate (*p* = 3.58 × 10^−6^) levels in post-COVID and control class were evaluated through multiple linear models and cluster-robust variance was used to estimate the standard error. All models showed no statistically significant effect of the considered covariates.

NMR data before rehabilitation were integrated with clinical parameters generating a correlation map with hierarchical clustering analysis (HCA) with R software^92^. Clinical test values and selected bin integrals of significant metabolites were combined using Pearson correlation as the distance metric. The Euclidean distance was considered for the metrics and the centroid method for clustering criterion.

Clinical parameters discriminating post-COVID-19 patients at the admission (*in*) and at discharge (*out*) after rehabilitation were evaluated by analyzing paired data with multilevel PLS-DA^93^ using the R software and the mixOmics package^94^. The paired samples Wilcoxon test was used to assess statistical significance.

### Network analysis

Enrichment analysis on metabolites from the post-COVID-19 *vs.* controls model was applied using the *diffusion* method computed with the FELLA package in R^95^. The *Homo sapiens* database in the Kyoto Encyclopedia of Genes and Genomes (KEGG)^96^ was used. The resulting network and subnetwork were evaluated with a threshold of *p* < 0.001. The results are reported in Supporting Table S1.

### miRNet in silico analysis

With the miRNet tool^97^ we predicted gene-modulated miRNAs. Genes were identified by gene-metabolite interaction network analysis that uncovered interactions between metabolites and genes^98^. Detailed implementation resources for miRNA-target data derived from miRTarBase v7.0, TarBase v7.0 and miRecords databases. Putative miRNA functions were identified using the hypergeometric test. Network size and complexity were reduced using miRNA-Function, the database for functional enrichment analysis. miRNAs functional implications were uncovered by using Tam2^99^. Gene Ontology and GO annotation data were obtained with QuickGO^100^.

### RNA isolation and quantitative real time PCR (qRT-PCR) miRNAs validation

After controlling for age (airway miRNAs may be age-dependent^101^), total RNA was extracted from *ca.* 1 mL of EBC from 20 subjects (10 healthy controls and 10 post-COVID-19 patients), which offered sufficient power to assess twofold changes. The purification kit (NorgenBiotek Corporation, Thorold, ON, Canada) was used according to the manufacturer’s instructions. Quantity and quality were analyzed by NanoDrop spectrophotometer (Thermo Fisher Scientific, Monza, MB, Italy), and subsequently stored at − 80 °C until use. The quantity of total RNA in each sample ranged from 3 to 11 ng/μL and was used in agreement with the transcription kit protocol. Exogenous spike-in, cel‐has-miR‐39‐3p, was added in a standardized amount to all samples prior to the RNA extraction to allow for normalization of technical variability. Furthermore, it was possible to estimate the degree of purity of RNA as a function of contamination from complex carbohydrates and proteins. For good RNA preparations, the A260/A280 purity ratios must be 1.8–2.0, as observed for all our samples. When this ratio is lower, it indicates the presence of contaminants (phenol or proteins absorbing near 280 nm). But, in this study, we specifically selected this procedure phenol‐free and with several filtering steps (removing larger particles) to obtain a total RNA pure extract. In addition, the Norgen kit protocol has been created also for biofluids and without any need of modifications for our EBC samples. Isolated RNA was used to synthesize cDNA using a reverse transcription kit (Applied Biosystems, Foster City, CA, USA).

Selected human miRNA (hsa-miR-34a-5p; hsa-miR-146a-5p; hsa-miR-126-3p; hsa-miR-223-3p; cel-hsa-miR-39-3p) expressions were quantified using the TaqMan MicroRNA assay (Applied Biosystems, Foster City, CA, USA), and qRT-PCR was performed on an ABI Prism 7500 Real Time PCR System (Applied Biosystems, Foster City, CA, USA). miRNAs are reported as relative expression normalized to the mean of a synthetic spiked-in non-human cel-hsa-miR-39-3p (5′-UCACCGGGUGUAAAUCAGCUUG; Life Technologies Europe BV, Bleiswijk, the Netherlands). The relative expression of each miRNA was reported as 2^−ΔCt^, with ΔCt being the difference between the Cts of the specific miRNA and those of the cel-hsa-miR-39-3p. Each reaction was performed in triplicate.

### Supplementary Information

Supplementary Information.

## Data Availability

All data associated with this study are present in the paper or the Supplementary Information.

## References

[1] Wu Z, McGoogan JM (2020). Characteristics of and important lessons from the coronavirus disease 2019 (COVID-19) outbreak in China: Summary of a report of 72314 cases from the Chinese Center for Disease Control and Prevention. JAMA.

[2] National Institute for Health and Care Excellence, Royal College of General Practitioners, Healthcare Improvement Scotland SIGN (2020). COVID-19 Rapid Guideline: Managing the Long-Term Effects of COVID-19.

[3] Logue, J. K. *et al*. Sequelae in adults at 6 months after COVID-19 infection. *JAMA Netw. Open* **4**, e210830 (2021). Erratum in: *JAMA Netw. Open* **4**, e214572 (2021). 10.1001/jamanetworkopen.2021.0830 PMC7896197 33606031

[4] Mizrahi B (2023). Long covid outcomes at one year after mild SARS-CoV-2 infection: Nationwide cohort study. BMJ.

[5] Global Burden of Disease Long COVID Collaborators (2022). Estimated global proportions of individuals with persistent fatigue, cognitive, and respiratory symptom clusters following symptomatic COVID-19 in 2020 and 2021. JAMA.

[6] Thaweethai T (2023). Development of a definition of postacute sequelae of SARS-CoV-2 infection. JAMA.

[7] Ballering AV (2022). Persistence of somatic symptoms after COVID-19 in the Netherlands: An observational cohort study. Lancet.

[8] Gottlieb M (2023). Long COVID clinical phenotypes up to 6 months after infection identified by latent class analysis of self-reported symptoms. Open Forum Infect. Dis..

[9] Davis, H. E., McCorkell, L., Vogel, J. M. & Topol, E. J. Long COVID: Major findings, mechanisms and recommendations. *Nat. Rev. Microbiol.* **21**, 133‒146 (2023). Erratum in: *Nat. Rev. Microbiol.* **21**, 408 (2023). 10.1038/s41579-022-00846-2 PMC9839201 36639608

[10] Goërtz YMJ (2023). Symptoms and quality of life before, during, and after a SARS-CoV-2 PCR positive or negative test: Data from lifelines. Sci. Rep..

[11] Demko ZO (2022). Post-acute sequelae of SARS-CoV-2 (PASC) impact quality of life at 6, 12 and 18 months post-infection. MedRxiv.

[12] Bach, K. Is ‘long Covid’ worsening the labor shortage? *Brookings.* https://www.brookings.edu/research/is-long-covid-worsening-the-labor-shortage/ (2022).

[13] Williamson AE, Tydeman F, Miners A, Pyper K, Martineau AR (2022). Short-term and long-term impacts of COVID-19 on economic vulnerability: A population-based longitudinal study (COVIDENCE UK). BMJ Open.

[14] Su Y (2022). Multiple early factors anticipate post-acute COVID-19 sequelae. Cell.

[15] Parker AM (2021). Addressing the post-acute sequelae of SARS-CoV-2 infection: A multidisciplinary model of care. Lancet Respir. Med..

[16] Greenhalgh T (2020). Management of post-acute covid-19 in primary care. BMJ.

[17] Maniscalco M (2019). Clinical metabolomics of exhaled breath condensate in chronic respiratory diseases. Adv. Clin. Chem..

[18] Singh SJ (2023). Respiratory sequelae of COVID-19: Pulmonary and extrapulmonary origins, and approaches to clinical care and rehabilitation. Lancet Respir. Med..

[19] Kovacic P, Somanathan R (2009). Pulmonary toxicity and environmental contamination: Radicals, electron transfer, and protection by antioxidants. Rev. Environ. Contam. Toxicol..

[20] Ahmed N (2016). Metabolic signatures of lung cancer in sputum and exhaled breath condensate detected by ^1^H magnetic resonance spectroscopy: A feasibility study. Magn. Reson. Insights.

[21] Vinolo MA (2011). Suppressive effect of short chain fatty acids on production of proinflammatory mediators by neutrophils. J. Nutr. Biochem..

[22] Wolak JE (2009). Metabolomic analysis of bronchoalveolar lavage fluid from cystic fibrosis patients. Biomarkers.

[23] Gong Y (2017). Blockage of glycolysis by targeting PFKFB3 alleviates sepsis-related acute lung injury via suppressing inflammation and apoptosis of alveolar epithelial cells. Biochem. Biophys. Res. Commun..

[24] Vassiliou AG (2020). Lactate kinetics reflects organ dysfunction and are associated with adverse outcomes in intensive care unit patients with COVID-19 pneumonia: Preliminary results from a Greek single-centre study. Metabolites.

[25] RECOVERY Collaborative Group (2021). Tocilizumab in patients admitted to hospital with COVID-19 (RECOVERY): A randomised, controlled, open-label, platform trial. Lancet.

[26] Ghini V (2022). Profiling metabolites and lipoproteins in COMETA, an Italian cohort of COVID-19 patients. PLoS Pathog..

[27] Maniscalco M (2022). Metabolomics of COPD pulmonary rehabilitation outcomes via exhaled breath condensate. Cells.

[28] Pieters M (2010). The effect of ethanol and its metabolism on fibrinolysis. Thromb. Haemost..

[29] Meoni G (2021). Metabolomic/lipidomic profiling of COVID-19 and individual response to tocilizumab. PLoS Pathog..

[30] Julkunen H (2021). Metabolic biomarker profiling for identification of susceptibility to severe pneumonia and COVID-19 in the general population. Elife.

[31] Bizkarguenaga M (2022). Uneven metabolic and lipidomic profiles in recovered COVID-19 patients as investigated by plasma NMR metabolomics. NMR Biomed..

[32] Del Valle DM (2020). An inflammatory cytokine signature predicts COVID-19 severity and survival. Nat. Med..

[33] Youm YH (2015). The ketone metabolite β-hydroxybutyrate blocks NLRP3 inflammasome-mediated inflammatory disease. Nat. Med..

[34] Bradshaw PC (2020). COVID-19: Proposing a ketone-based metabolic therapy as a treatment to blunt the cytokine storm. Oxid. Med. Cell. Longev..

[35] Bruzzone C (2020). SARS-CoV-2 infection dysregulates the metabolomic and lipidomic profiles of serum. iScience.

[36] Maurice DH (2014). Advances in targeting cyclic nucleotide phosphodiesterases. Nat. Rev. Drug Discov..

[37] Khani E (2021). Potential pharmacologic treatments for COVID-19 smell and taste loss: A comprehensive review. Eur. J. Pharmacol..

[38] Ang Z (2018). FFAR2-FFAR3 receptor heteromerization modulates short-chain fatty acid sensing. FASEB J..

[39] Zaid Y (2021). Chemokines and eicosanoids fuel the hyperinflammation within the lungs of patients with severe COVID-19. J. Allergy Clin. Immunol..

[40] Jaffal SM, Abbas MA (2021). TRP channels in COVID-19 disease: Potential targets for prevention and treatment. Chem. Biol. Interact..

[41] Scheraga RG (2020). The role of TRPV4 in regulating innate immune cell function in lung inflammation. Front. Immunol..

[42] Dutta Banik D (2018). TRPM4 and TRPM5 are both required for normal signaling in taste receptor cells. Proc. Natl. Acad. Sci. U.S.A..

[43] Baxter BD (2021). Transcriptional profiling reveals potential involvement of microvillous TRPM5-expressing cells in viral infection of the olfactory epithelium. BMC Genom..

[44] Watanabe H (2008). TRP channel and cardiovascular disease. Pharmacol. Ther..

[45] Liang Y (2023). Circulating microRNAs as emerging regulators of COVID-19. Theranostics.

[46] Ahmad W (2023). Differentially-regulated miRNAs in COVID-19: A systematic review. Rev. Med. Virol..

[47] Widiasta A (2020). Potential role of ACE2-related microRNAs in COVID-19-associated nephropathy. Noncoding RNA Res..

[48] Yang HH (2017). Protective effects of microRNA-126 on human cardiac microvascular endothelial cells against hypoxia/reoxygenation-induced injury and inflammatory response by activating PI3K/Akt/eNOS signaling pathway. Cell Physiol. Biochem..

[49] Mitchell MI (2021). Extracellular vesicle capture by AnTibody of CHoice and enzymatic release (EV-CATCHER): A customizable purification assay designed for small-RNA biomarker identification and evaluation of circulating small-EVs. J. Extracell. Vesicles.

[50] Keikha R (2021). The relative expression of miR-31, miR-29, hsa-miR-126-3p, and miR-17 and their mRNA targets in the serum of COVID-19 patients with different grades during hospitalization. Eur. J. Med. Res..

[51] Nicoletti AS (2022). Differentially expressed plasmatic microRNAs in Brazilian patients with Coronavirus disease 2019 (COVID-19): Preliminary results. Mol. Biol. Rep..

[52] Grehl C (2021). Detection of SARS-CoV-2 derived small RNAs and changes in circulating small RNAs associated with COVID-19. Viruses.

[53] Sabbatinelli J (2021). Decreased serum levels of the inflammaging marker hsa-miR-146a-5p are associated with clinical non-response to tocilizumab in COVID-19 patients. Mech. Ageing Dev..

[54] Schober A (2014). MicroRNA-126-5p promotes endothelial proliferation and limits atherosclerosis by suppressing Dlk1. Nat. Med..

[55] Ambrosino P (2021). Persistent endothelial dysfunction in post-acute COVID-19 syndrome: A case-control study. Biomedicines.

[56] Wang Y (2021). Decreased inhibition of exosomal miRNAs on SARS-CoV-2 replication underlies poor outcomes in elderly people and diabetic patients. Signal Transduct. Target Ther..

[57] Neudecker V (2017). Myeloid-derived hsa-miR-223-3p regulates intestinal inflammation via repression of the NLRP3 inflammasome. J. Exp. Med..

[58] Houshmandfar S (2021). miRNA-223 as a regulator of inflammation and NLRP3 inflammasome, the main fragments in the puzzle of immunopathogenesis of different inflammatory diseases and COVID-19. Naunyn Schmiedebergs Arch. Pharmacol..

[59] Mo R, Li J, Chen Y, Ding Y (2022). lncRNA GAS5 promotes pyroptosis in COPD by functioning as a ceRNA to regulate the miR-223-3p/NLRP3 axis. Mol. Med. Rep..

[60] Meidert AS (2021). Extracellular vesicle associated miRNAs regulate signaling pathways involved in COVID-19 Pneumonia and the progression to severe acute respiratory corona virus-2 syndrome. Front. Immunol..

[61] Rasizadeh R (2023). SARS-CoV-2-associated organs failure and inflammation: A focus on the role of cellular and viral microRNAs. Virol. J..

[62] Arghiani N, Nissan T, Matin MM (2021). Role of microRNAs in COVID-19 with implications for therapeutics. Biomed. Pharmacother..

[63] Griffiths-Jones S (2004). The microRNA registry. Nucleic Acids Res..

[64] Zhang S (2021). The miRNA: A small but powerful RNA for COVID-19. Brief. Bioinform..

[65] Donyavi T (2021). Acute and post-acute phase of COVID-19: Analyzing expression patterns of miRNA-29a-3p, 146a–3p, 155–5p, and let-7b-3p in PBMC. Int. Immunopharmacol..

[66] Tang H (2020). The noncoding and coding transcriptional landscape of the peripheral immune response in patients with COVID-19. Clin. Transl. Med..

[67] Nie X (2021). Multi-organ proteomic landscape of COVID-19 autopsies. Cell.

[68] Henriques-Pons A (2022). Pulmonary mesenchymal stem cells in mild cases of COVID-19 are dedicated to proliferation; in severe cases, they control inflammation, make cell dispersion, and tissue regeneration. Front. Immunol..

[69] Thompson EA (2021). Metabolic programs define dysfunctional immune responses in severe COVID-19 patients. Cell Rep..

[70] Cunha LL (2020). Remodeling of the immune response with aging: Immunosenescence and its potential impact on COVID-19 immune response. Front. Immunol..

[71] Fülöp T (2021). Immunology of aging: The birth of inflammaging. Clin. Rev. Allergy Immunol..

[72] Wilk AJ (2021). Multi-omic profiling reveals widespread dysregulation of innate immunity and hematopoiesis in COVID-19. J. Exp. Med..

[73] Hunter, C. A. & Jones, S. A. IL-6 as a keystone cytokine in health and disease. *Nat. Immunol.* **16**, 448‒457 (2015). Erratum in: *Nat. Immunol.* **18**, 1271 (2017). 10.1038/ni.3153 25898198

[74] Ackermann M (2020). Pulmonary vascular endothelialitis, thrombosis, and angiogenesis in Covid-19. N. Engl. J. Med..

[75] Ackermann M (2020). Inflammation and intussusceptive angiogenesis in COVID-19: Everything in and out of flow. Eur. Respir. J..

[76] Bloom PP (2021). Liver biochemistries in hospitalized patients with COVID-19. Hepatology.

[77] Papic N (2012). Liver involvement during influenza infection: Perspective on the 2009 influenza pandemic. Influenza Other Respir. Viruses.

[78] Marjot T (2021). COVID-19 and liver disease: Mechanistic and clinical perspectives. Nat. Rev. Gastroenterol. Hepatol..

[79] Al-Samkari H (2020). COVID-19 and coagulation: Bleeding and thrombotic manifestations of SARS-CoV-2 infection. Blood.

[80] Vidali S (2020). D-dimer as an indicator of prognosis in SARS-CoV-2 infection: A systematic review. ERJ Open Res..

[81] von Elm E (2007). The Strengthening the Reporting of Observational Studies in Epidemiology (STROBE) statement: Guidelines for reporting observational studies. Lancet.

[82] Miller MR (2005). Standardisation of spirometry. Eur. Respir. J..

[83] Macintyre N (2005). Standardisation of the single-breath determination of carbon monoxide uptake in the lung. Eur. Respir. J..

[84] Jones PW (2009). Development and first validation of the COPD assessment test. Eur. Respir. J..

[85] Enright PL, Sherrill DL (1998). Reference equations for the six-minute walk in healthy adults. Am. J. Respir. Crit. Care Med..

[86] Adamo S (2022). A machine learning approach to predict the rehabilitation outcome in convalescent COVID-19 patients. J. Pers. Med..

[87] Paris D (2018). Nuclear magnetic resonance-based metabolomics in respiratory medicine. Eur. Respir. J..

[88] Maniscalco M (2020). Metabolomics of exhaled breath condensate by nuclear magnetic resonance spectroscopy and mass spectrometry: A methodological approach. Curr. Med. Chem..

[89] Maniscalco M (2017). Coexistence of obesity and asthma determines a distinct respiratory metabolic phenotype. J. Allergy Clin. Immunol..

[90] Eriksson L, Byrne T, Johansson E, Trygg J, Wikström C (2013). Multi and Megavariate Data Analysis. Part I: Basic Principles and Applications.

[91] Stuart E, King G, Imai K, Ho D (2011). MatchIT: Nonparametric preprocessing for parametric causal inference. J. Stat. Softw..

[92] R Core Team. *R: A Language and Environment for Statistical Computing*. www.R-project.org/ (R Foundation for Statistical Computing, 2021).

[93] Westerhuis JA (2010). Multivariate paired data analysis: Multilevel PLSDA versus OPLSDA. Metabolomics.

[94] Lê Cao, K. A. *et al*. *mixOmics: Omics Data Integration Project. R Package Version 6.1.1*. https://CRAN.R-project.org/package=mixOmics (2016).

[95] Picart-Armada S (2018). FELLA: An R package to enrich metabolomics data. BMC Bioinform..

[96] Kanehisa M (2023). KEGG for taxonomy-based analysis of pathways and genomes. Nucleic Acids Res..

[97] Fan Y (2016). miRNet—Dissecting miRNA–target interactions and functional associations through network-based visual analysis. Nucleic Acids Res..

[98] Pang ZQ (2021). MetaboAnalyst 5.0: Narrowing the gap between raw spectra and functional insights. Nucleic Acids Res..

[99] Li J (2018). TAM 2.0: Tool for microRNA set analysis. Nucleic Acids Res..

[100] Binns D (2009). QuickGO: A web-based tool for Gene Ontology searching. Bioinformatics.

[101] Ong J (2019). Age-related gene and miRNA expression changes in airways of healthy individuals. Sci. Rep..

