# Supplementary material for: The biomarkers’ landscape of post-COVID-19 patients can suggest selective clinical interventions
Source: Sci Rep. 2023 Dec 15;13:22496. doi: 10.1038/s41598-023-49601-4 (PMC10728085; doi:10.1038/s41598-023-49601-4)
Supplement: Supplementary file 1 — Supplementary Information. [file 41598_2023_49601_MOESM1_ESM.docx]

**Supplementary Information for**

**The biomarkers’ landscape of post-COVID-19 patients can suggest selective clinical interventions**

Debora Paris^1^, Letizia Palomba^2^, Maria Cristina Albertini^2^, Annabella Tramice^1^, Lorenzo Motta^3†^, Eleonora Giammattei^2^, Pasquale Ambrosino^4^, Mauro Maniscalco^5,6^*, Andrea Motta^1,^*

^1^Institute of Biomolecular Chemistry, National Research Council; 80078 Pozzuoli (Naples), Italy.

^2^Department of Biomolecular Sciences, “Carlo Bo” University; 61029 Urbino, Italy.

^3^Ospedale Santa Maria della Misericordia, Neuroradiology Unit, 45100 Rovigo, Italy.

^4^Istituti Clinici Scientifici Maugeri IRCCS, Directorate of Telese Terme Institute; 82037 Telese Terme (Benevento), Italy.

^5^Istituti Clinici Scientifici Maugeri IRCCS, Pulmonary Rehabilitation Unit of the Telese Terme Institute; 82037 Telese Terme (Benevento), Italy.

^6^Department of Clinical Medicine and Surgery, Section of Respiratory Disease, University of Naples Federico II; 80131 Naples, Italy.

†Current address: IRCCS Istituto delle Scienze Neurologiche (padiglione G), via Altura 3, 40139 Bologna, Italy.

*Email: [**andrea.motta@icb.cnr.it**](mailto:andrea.motta@icb.cnr.it); [**mauro.maniscalco@icsmaugeri.it**](mailto:mauro.maniscalco@icsmaugeri.it) or [**mauro.maniscalco@unina.it**](mailto:mauro.maniscalco@unina.it)

**Materials and methods**

*Patients*

Convalescent COVID-19 patients referring to the Pulmonary Rehabilitation Unit of Istituti Clinici Scientifici Maugeri IRCCS, Telese Terme, Italy, were screened for enrollment within 2 months of swab test negativization from October 2020 to February 2021. Inclusion criteria were: age ≥18 years; recent SARS-CoV-2 infection with severe-to-critical COVID-19 according to the NIH classification [<https://www.covid19treatmentguidelines.nih.gov/overview/clinical-spectrum/>]; persistent clinical or functional manifestations after a negative swab test; indication to start a multidisciplinary rehabilitation program. Exclusion criteria were: recent (<6 months) major surgery or any previous lung surgery; current malignancy; any history of chronic respiratory disease (*e.g.*, asthma, chronic obstructive pulmonary disease) other than COVID-19; inability to understand or sign the informed consent. A group of age- and sex-matched non-COVID-19 controls was selected from an historical cohort of volunteer subjects undergoing EBC collection. Participants with missing data for the outcome of interest were excluded from the study.

This protocol was performed following the Strengthening the Reporting of Observational Studies in Epidemiology (STROBE) reporting guidelines^1^, in line with the 1975 Declaration of Helsinki. The Ethic Committee of Istituto Nazionale Tumori, Fondazione Pascale, Naples, Italy approved the study with reference number ICS 3/20. All study participants provided written informed consent.

*Study procedures*

After signing the informed consent, all convalescent COVID-19 patients underwent a detailed collection of key demographic and clinical information related to the acute phase of COVID-19, lung function, physical performance, comorbidities and treatments in course. Clinical data and EBC samples from age- and sex-matched healthy volunteers were also included in the study as controls. They belonged to an irreversible deidentified set of electronic Maugeri database containing records of people selected from the hospital staff, whose samples (including EBC) were previously collected and stored at -80°C. The absence of significant respiratory, cardiac and/or metabolic diseases were anamnestic. Venous blood samples for the common hemato-chemical parameters were collected at the admission and at the end of rehabilitation. Arterial blood samples were collected to measure oxygen (PaO_2_) and carbon dioxide tension (PaCO_2_) using a blood gas analyzer (ABL 825® FLEX BGA, Radiometer Medical Aps, Copenhagen, Denmark). According to the protocols of the American Thoracic Society and the European Respiratory Society^2,3^, spirometry parameters and diffusion lung capacity for carbon monoxide (DLCO) were also evaluated with an automated equipment (Vmax® Encore, Vyasis Healthcare, Milan, Italy). Forced expiratory volume in 1 second (FEV_1_), forced vital capacity (FVC) and DLCO were expressed both as numerical values and percentages of predicted values (FEV_1_%, FVC% and DLCO%, respectively). The chronic obstructive pulmonary disease (COPD) Assessment Test (CAT)^4^ and the Barthel index were also administered to all post COVID-19 patients to determine the impact of the disease on the level of functioning and activities of daily living. Exercise capacity was tested by measuring the six-minute walking distance (6MWD)^5^. All the instrumental analyses were carried out at the admission (in) and at the discharge (out) after rehabilitation.

*Rehabilitation*

The rehabilitation protocol applied in our Pulmonary Rehabilitation Unit follows the official ATS/ERS guidelines and it has been detailed elsewhere^6^. In brief, all study participants underwent a 5-week exercise-based program of 6 sessions/week (30 sessions), with dietary and psychosocial counselling. Physical exercise was the cornerstone of the program, based on treadmill walking, stationary cycling, arm ergometry, flexibility, stretching and strengthening exercises with body and fixed weights. A physiotherapist monitored and supervised participation.

*EBC collection*

EBC samples were collected from negativized patients (post-COVID) before entering the rehabilitation program. All subjects refrained from food intake for 8 hours before the test and from alcoholic drinks for 18 hours. EBC was collected from patients in random order and in the same room with a TURBO-DECCS condenser (Medivac, Pilastrello, Parma, Italy, [www.medivac.it](file:///E:\-IN%20PROGRESS\Post-COVID19\MANOSCRITTO\PostCOVID%20Finale\www.medivac.it)) set at -5.0±1.0°C as reported^7,8^. On average, from each subject we collected 2.0±0.4 ml (mean±SD) of EBC. With a 3-min gentle nitrogen gas flow we removed volatile substances. Samples were immediately stored at −80°C until NMR acquisition.

*NMR sample preparation and spectra acquisition*

To 630 μl of EBC we added 70 μl of a ^2^H_2_O solution [containing 0.1 mmol/l 3-(Trimethylsilyl)propionic-2,2,3,3-d4 acid sodium salt (TSP) for ^1^H chemical shift reference and 3 mmol/l sodium azide]. NMR spectra were recorded on a Bruker Avance III 600-MHz spectrometer equipped with a CryoProbe and an automatic and cooled sample changer of 24 positions (Bruker BioSpin GmbH, Rheinstetten, Germany). Sample temperature was kept constant at 300 K (27°C). 1D spectra including water suppression with excitation sculpting sequence, and homo- and heteronuclear 2D experiments (^1^H−^1^H clean TOCSY and ^1^H-^13^C HSQC) were acquired as previously described^7,8^. In particular, for 1D spectra, a double–pulsed field gradient echo, with a soft square pulse of 4 ms at the water resonance frequency, with the gradient pulse of 1 ms each in duration, adding 256 transients of 16384 points with a spectral width of 8389.3 Hz (which corresponds to 14 ppm) was used. Time-domain data were next zero-filled to 32768 points, and prior to Fourier transformation, an exponential multiplication of 0.6 Hz was applied. A typical EBC spectrum of post-COVID is shown in Fig. 1S.

*Power analysis*

*A priori* power analysis in metabolomics is not feasible because concentration variations of biomarkers are not known before analysis^9^. For an estimation, we varied the 1-α and 1-β parameters from 95% to 99.9% and from 80% to 99.9%, respectively. Using the accuracy percentages obtained in our validation tests for a 1-α value of 95% and a 1-β value of 80%^9^, we derived 24 ± 3 post-COVID-19 patients for all classes, while for 1-α = 1-β = 99.9% we obtained 28 ± 3 patients. To account for possible drop-outs or protocol adherence problems, we screened 60 post-COVID-19 patients, with the final number of enrolled patients (40) greater than that indicated by the backward analysis. However, in common practice, 1-α = 95% and 1-β = 80%, and 99.9% is an extreme setting.

*Multivariate data analysis*

EBC proton spectra ranging from 9.0 to 0.60 ppm were automatically subdivided into 420 discrete regions (‘buckets’) of equal width (Δδ= 0.02 ppm) and integrated using the AMIX 3.9.15 software package (Bruker BioSpin GmbH, Rheinstetten, Germany). We left out from the analysis the 5.0-4.6 ppm region around the water resonance, and each integral was normalized to the total spectrum area to account for possible dilution effects on signals. NMR data were arranged into a matrix (X matrix) and then imported into SIMCA-P+14 package (Umetrics, Umeå, Sweden) where Principal Components Analysis (PCA) and Orthogonal Projections to Latent Structures Discriminant Analysis (OPLS-DA) were performed, after Pareto scaling data pretreatment. Firstly, PCA was applied to reduce data dimensionality thus obtaining an overview of data structure. With such unsupervised analysis, we explored possible trends and checked for potential outliers. Once class homogeneity was assessed for post-COVID patients and healthy subjects, supervised OPLS-DA was applied to search for clustering and discrimination. For this purpose, a Y matrix was created assigning dummy variables to each EBC sample in order to define its class belonging. Supervised discriminant regressions were then conducted between X and Y matrix, thus generating predictive models that better relate metabolites variation to each EBC class. In this context, comparing patients before starting their rehabilitation therapy (post-COVID group) with age-matched healthy subjects (non-COVID group) was useful to investigate the effect of sequelae of SARS-CoV-2 infection. Model quality was evaluated by using the goodness-of-fit parameter (R^2^) and the goodness-of-prediction parameter (Q^2^)^10^, together with an internal iterative 7-round cross-validation and permutation test (800 repeats) and ANalysis Of VAriance testing of Cross-Validated predictive residuals (CV-ANOVA). Quantification was achieved with OriginPro 9.1 software package (OriginLab Corporation, Northampton, USA). Statistical significance for selected metabolites was determined by parametric (ANOVA with Bonferroni correction) or non-parametric (Mann-Whitney U) tests according to the results of normality test performed to evaluate data distribution (Shapiro-Wilk, Kolgomorov-Smirnov test). *p* values < 0.05 were considered statistically significant.

To assess the post-COVID-19 metabolic state before rehabilitation, spectroscopic data (X matrix) were integrated with clinical parameters (Z matrix). For this purpose, a correlation map with hierarchical clustering analysis (HCA) was generated with R software^11^ by combining clinical test values and selected bin integrals of significant metabolites using Pearson correlation. The Euclidean distance was considered for the metrics and the centroid method for clustering criterion.

Clinical parameters discriminating post-COVID-19 patients at the admission (*in*) and at discharge (*out*) after rehabilitation were evaluated by analyzing paired data with multilevel PLS-DA^12^ using the R software and the mixOmics package^13^.

*Network analysis*

Enrichment analysis on selected and more representative metabolites found in post-COVID and non-COVID class separation was applied using the ‘diffusion’ method computed with the FELLA package in R^14^. Starting from the set of altered compounds, such analysis suggested affected reactions, enzymes, modules and pathways using label propagation in a knowledge model network based on *Homo sapiens* database in the Kyoto Encyclopedia of Genes and Genomes (KEGG)^15^. The resulting network and subnetwork were reported with a threshold of *p*< 0.05 in Table S2.

*miRNet in silico analysis*

The miRNet tool^16^ was used to predict gene-modulated microRNAs. Involved genes were identified by gene-metabolite interaction network analysis that predicted interactions between functionally related metabolites and genes^17^. Detailed implementation resources for miRNA-target data derived from miRTarBase v7.0, TarBase v7.0 and miRecords databases. We used the hypergeometric test to identify the putative miRNA functions obtained with our analysis. To reduce the network size and complexity and to retain the most relevant information for downstream functional analysis, we used miRNA-Function, the database for functional enrichment analysis, which yields *p* values and the number of hits. miRNAs functional implications were uncovered by using Tam2^18^. Gene Ontology and GO annotation data were obtained with QuickGO^19^.

*RNA isolation and quantitative Real Time PCR (qRT-PCR) microRNAs validation*

Since in the airways of healthy individuals’ miRNAs may be age-dependent^20^, analyses were conducted after controlling for age. Total RNA was extracted from *ca.* 1 ml of EBC from 20 subjects (10 healthy controls and 10 post-COVID-19 patients), which offered sufficient power to assess twofold changes, and was extracted by using an RNA purification kit (NorgenBiotek Corporation, Thorold, ON, Canada) according to the manufacturer’s instructions. RNA quantity and quality were analyzed by NanoDrop spectrophotometer (Thermo Fisher Scientific, Italy), and subsequently stored at −80 °C until use. Isolated RNA was used to synthesize cDNA using a reverse transcription kit (Applied Biosystems, Foster City, CA, USA) according to the manufacturer’s instructions.

Selected human miRNA (hsa-miR-34a-5p, hsa-miR-146a-5p, hsa-miR-126-3p and hsa-miR-223-3p) expressions were quantified using the TaqMan MicroRNA assay (Applied Biosystems, Foster City, CA, USA), and qRT-PCR was performed on an ABI Prism 7500 Real Time PCR System (Applied Biosystems, Foster City, CA, USA). miRNAs are reported as relative expression normalized to the mean of a synthetic spiked-in non-human cel-miR-39 (5′-UCACCGGGUGUAAAUCAGCUUG; Life Technologies Europe BV, Bleiswijk, the Netherlands). The relative expression of each miRNA was reported as 2^−ΔCt^, with ΔCt being the difference between the Cts of the specific miRNA and those of the cel-miR-39. Each reaction was performed in triplicate.

**References**

1. von Elm, E., et al. The Strengthening the Reporting of Observational Studies in Epidemiology (STROBE) statement: guidelines for reporting observational studies. *Lancet.* 370, 1453‒1457 (2007).

2. Miller, M. R., et al. Standardisation of spirometry. *Eur. Respir. J.* **26**, 319‒338 (2005).

3. Macintyre, N., et al. Standardisation of the single-breath determination of carbon monoxide uptake in the lung. *Eur. Respir. J.* **26**, 720‒735 (2005).

4. Jones, P. W., et al. Development and first validation of the COPD Assessment Test. *Eur. Respir. J.* **34**, 648‒654 (2009).

5. Enright, P. L., Sherrill, D. L. Reference equations for the six-minute walk in healthy adults. *Am. J. Respir. Crit. Care Med.* **158**, 1384‒1387 (1998).

6. Adamo, S., et al. A machine learning approach to predict the rehabilitation outcome in convalescent COVID-19 patients. *J. Pers. Med.* **12**, 328 (2022).

7. Paris, D., Maniscalco, M., Motta, A. Nuclear magnetic resonance-based metabolomics in respiratory medicine. *Eur. Respir. J.* **52**, 1801107 (2018).

8. Maniscalco, M., et al. Metabolomics of exhaled breath condensate by nuclear magnetic resonance spectroscopy and mass spectrometry: A methodological approach. *Curr. Med. Chem.* **27**, 2381‒2399 (2020).

9. Maniscalco, M., et al. Coexistence of obesity and asthma determines a distinct respiratory metabolic phenotype. *J. Allergy Clin. Immunol.* **139**, 1536‒1547 (2017).

10. Eriksson, L., Byrne, T., Johansson, E., Trygg, J., Wikström, C. *Multi and megavariate data analysis. Part I: Basic principles and applications* (3rd ed.). Umetrics AB (2013).

11. R Core Team. R: A language and environment for statistical computing. R Foundation for Statistical Computing, Vienna, Austria (2021). [www.R-project.org](http://www.R-project.org/)/.

12. Westerhuis, J. A., et al. Multivariate paired data analysis: multilevel PLSDA versus OPLSDA. *Metabolomics.* **6**, 119‒128 (2010).

13. Lê Cao, K.-A., et al. mixOmics: Omics Data Integration Project. R package version 6.1.1. (2016). <https://CRAN.R-project.org/package=mixOmics>.

14. Picart-Armada, S., et al. FELLA: an R package to enrich metabolomics data. *BMC Bioinformatics.* **19**, 538 (2018).

15. Kanehisa, M., et al. KEGG for taxonomy-based analysis of pathways and genomes. *Nucleic Acids Res.* **51**, D587‒D592 (2023).

16. Fan, Y. N., et al. miRNet - dissecting miRNA-target interactions and functional associations through network-based visual analysis. *Nucleic Acids Res.* **44**, W135‒W141 (2016).

17. Pang, Z. Q., et al. MetaboAnalyst 5.0: narrowing the gap between raw spectra and functional insights. *Nucleic Acids Res.* **49**, W388‒W396 (2021).

18. Li, J., et al. TAM 2.0: tool for MicroRNA set analysis. *Nucleic Acids Res.* **46**, W180‒W185 (2018).

19. Binns, D., et al. QuickGO: a web-based tool for Gene Ontology searching. *Bioinformatics.* 25, 3045‒3046 (2009).

20. Ong, J., et al. Age-related gene and miRNA expression changes in airways of healthy individuals. *Sci. Rep.* 9, 3765 (2019).

| **Table S1.** Metabolites assigned in EBC samples and corresponding ^1^H chemical shifts | | |
| --- | --- | --- |
| **Metabolites** | **Moieties** | **δ ^1^H (ppm) and multiplicity** |
| 2-Hydroxyisovalerate | αCH | 3.85 (d) |
|  | βCH | 2.02 (m) |
|  | γCH_3_ | 0.85 (d) |
|  | γ'CH_3_ | 0.98 (d) |
| 3-Hydroxyisobutyrate | αCH | 4.15 (m) |
|  | γCH_3_ | 1.06 (d) |
| 3-Hydroxyisovalerate | βCH_3_ | 1.25 (s) |
| Acetate | βCH_3_ | 1.90 (s) |
| Acetoin | CH | 4.42 (q) |
|  | CH_3_ | 2.21 (s) |
|  | CH_3_ | 1.38 (d) |
| Acetone | CH_3_ | 2.22 (s) |
| Alanine | βCH_3_ | 1.46 (d) |
|  | CH | 3.77 (m) |
| Benzoate | C_2,6_H, Ring | 7.86 (m) |
|  | C_3,5_H, Ring | 7.55 (m) |
|  | C_4_H, Ring | 7.47 (m) |
|  | CH_2_ | 3.03 (s) |
| Creatinine | N-CH_3_ | 4.03 (s) |
|  | CH_2_ | 3.03 (s) |
| Dimethylamine | CH_3_ | 2.73 (s) |
| Ethanol | CH_3_ | 1.17 (t) |
|  | CH_2_ | 3.64 (q) |
| Formate | HCOO^-^ | 8.45 (s) |
| Glycine | αCH_2_ | 3.54 (s) |
| Isobutyrate | βCH_3_ | 1.06 (d) |
|  | αCH | 2.38 (m) |
| Isocaproate | αCH_2_ | 2.20 (m) |
|  | βCH_2_ | 1.52(m) |
|  | γCH | 1.46(m) |
|  | δCH_3_ | 0.88 (d) |
| Isopropanol | CH | 4.02 (m) |
|  | (CH_3_)_2_ | 1.18 (d) |
| Lactate | βCH_3_ | 1.34 (d)) |
|  | αCH | 4.11 (q) |
| Lysine | ϵCH_2_ | 3.06 (t) |
| Methanol | CH_3_ | 3.34 (s) |
| Methylamine | CH_3_ | 2.60 (s) |
| N,N-Dimethylglycine | CH_3_ | 2.91 (s) |
|  | CH_2_ | 3.71 (s) |
| Ornithine | αCH | 3.77 (t) |
|  | δCH_3_ | 3.04 (t) |
| Phenylalanine | C_2,6_H, Ring | 7.37 (t) |
|  | C_3,5_H, Ring | 7.42 (t) |
|  | C_4_H, Ring | 7.37 (t) |
| Propionate | αCH_2_ | 2.19 (q) |
|  | βCH_3_ | 1.06 (t) |
| Pyruvate | CH_3_ | 2.36 (s) |
| Serine | αCH | 3.85 (dd) |
|  | βCH | 3.96 (dd) |
| Succinate | α,βCH_2_ | 2.42 (s) |
| Trimetylamine | CH_3_ | 2.91 (t) |
| Tyrosine | C_3,5_H, Ring | 6.88 (d) |
|  | C_2,6_H, Ring | 7.17 (d) |
| Valerate | αCH_2_ | 2.19 (t) |
|  | βCH_2_ | 1.52 (m) |
|  | γCH_2_ | 1.31 (m) |
|  | δCH_3_ | 0.90 (t) |
| Valine | αCH | 3.60 (d) |
|  | βCH | 2.26 (m) |
|  | γCH_3_ | 0.98 (d), |
|  | γ'CH_3_ | 1.03 (d) |

s = singlet; d = doublet; dd = double doublet; t = triplet; q = quartet; m = multiplet.

**Table S2**- Pathways, modules, enzymes, reactions and metabolites resulting from the enrichment analysis on discriminant metabolites responsible for post-COVID/Controls EBC class discrimination, using Homo sapiens database.

| KEGG id | Entry type | KEGG name (Homo sapiens) | *p* score |
| --- | --- | --- | --- |
| hsa00010 | pathway | Glycolysis / Gluconeogenesis | 2.97×10^-3^ |
| hsa00062 | pathway | Fatty acid elongation | 8.22×10^-4^ |
| hsa00072 | pathway | Synthesis and degradation of ketone bodies | 1.00×10^-6^ |
| hsa00620 | pathway | Pyruvate metabolism | 1.00×10^-6^ |
| hsa00640 | pathway | Propanoate metabolism | 1.41×10^-4^ |
| hsa00650 | pathway | Butanoate metabolism | 1.00×10^-6^ |
| hsa04024 | pathway | cAMP signaling pathway | 7.81×10^-4^ |
| hsa04061 | pathway | Viral protein interaction with cytokine and cytokine receptor | 4.63×10^-2^ |
| hsa04152 | pathway | AMPK signaling pathway | 3.81×10^-2^ |
| hsa04213 | pathway | Longevity regulating pathway - multiple species | 5.30×10^-3^ |
| hsa04750 | pathway | Inflammatory mediator regulation of TRP channels | 9.29×10^-3^ |
| hsa04911 | pathway | Insulin secretion | 1.78×10^-6^ |
| hsa04920 | pathway | Adipocytokine signaling pathway | 7.03×10^-4^ |
| hsa04930 | pathway | Type II diabetes mellitus | 1.00×10^-6^ |
| hsa04932 | pathway | Non-alcoholic fatty liver disease | 1.00×10^-6^ |
| hsa05014 | pathway | Amyotrophic lateral sclerosis | 3.23×10^-2^ |
| hsa05034 | pathway | Alcoholism | 1.00×10^-6^ |
| M00027 | module | GABA (gamma-Aminobutyrate) shunt | 3.48×10^-2^ |
| M00088 | module | Ketone body biosynthesis, acetyl-CoA => acetoacetate/3-hydroxybutyrate/acetone | 1.00×10^-6^ |
| M00151 | module | Cytochrome bc1 complex respiratory unit | 2.54×10^-2^ |
| M00152 | module | Cytochrome bc1 complex | 2.54×10^-2^ |
| M00168 | module | CAM (Crassulacean acid metabolism), dark | 1.94×10^-3^ |
| M00169 | module | CAM (Crassulacean acid metabolism), light | 6.32×10^-4^ |
| M00172 | module | C4-dicarboxylic acid cycle, NADP - malic enzyme type | 2.21×10^-3^ |
| 1.1.1.2 | enzyme | alcohol dehydrogenase (NADP+) | 2.95×10^-3^ |
| 1.1.1.27 | enzyme | L-lactate dehydrogenase | 1.84×10^-2^ |
| 1.1.1.30 | enzyme | 3-hydroxybutyrate dehydrogenase | 2.71×10^-3^ |
| 1.1.1.38 | enzyme | malate dehydrogenase (oxaloacetate-decarboxylating) | 8.79×10^-4^ |
| 1.1.1.40 | enzyme | malate dehydrogenase (oxaloacetate-decarboxylating)(NADP+) | 1.77×10^-2^ |
| 1.1.2.4 | enzyme | D-lactate dehydrogenase (cytochrome) | 1.13×10^-3^ |
| 1.11.1.6 | enzyme | catalase | 1.00×10^-6^ |
| 1.3.5.1 | enzyme | succinate dehydrogenase | 2.59×10^-2^ |
| 2.3.3.10 | enzyme | hydroxymethylglutaryl-CoA synthase | 6.05×10^-3^ |
| 2.7.10.1 | enzyme | receptor protein-tyrosine kinase | 4.63×10^-2^ |
| 2.7.11.17 | enzyme | Ca2+/calmodulin-dependent protein kinase | 1.89×10^-2^ |
| 3.1.1.23 | enzyme | acylglycerol lipase | 2.90×10^-6^ |
| 3.1.1.3 | enzyme | triacylglycerol lipase | 1.09×10^-4^ |
| 3.1.1.32 | enzyme | phospholipase A1 | 1.11×10^-2^ |
| 3.1.1.34 | enzyme | lipoprotein lipase | 1.46×10^-3^ |
| 3.1.1.5 | enzyme | lysophospholipase | 3.49×10^-4^ |
| 3.1.1.56 | enzyme | methylumbelliferyl-acetate deacetylase | 1.00×10^-6^ |
| 3.1.1.64 | enzyme | retinoid isomerohydrolase | 2.55×10^-2^ |
| 3.1.1.7 | enzyme | acetylcholinesterase | 2.67×10^-2^ |
| 3.1.2.1 | enzyme | acetyl-CoA hydrolase | 1.00×10^-6^ |
| 3.5.1.23 | enzyme | ceramidase | 1.04×10^-3^ |
| 3.5.1.89 | enzyme | N-acetylglucosaminylphosphatidylinositol deacetylase | 1.00×10^-6^ |
| 3.5.1.98 | enzyme | histone deacetylase | 3.87×10^-3^ |
| 4.1.3.4 | enzyme | hydroxymethylglutaryl-CoA lyase | 1.00×10^-6^ |
| 6.2.1.16 | enzyme | acetoacetate---CoA ligase | 9.62×10^-3^ |
| 6.4.1.2 | enzyme | acetyl-CoA carboxylase | 3.15×10^-2^ |
| 7.1.1.2 | enzyme | NADH:ubiquinone reductase (H+-translocating) | 3.55×10^-2^ |
| 7.1.1.8 | enzyme | quinol---cytochrome-c reductase | 1.74×10^-2^ |
| R00009 | reaction | hydrogen-peroxide:hydrogen-peroxide oxidoreductase | 1.35×10^-2^ |
| R00152 | reaction | hydrogen cyanide aminohydrolase | 4.67×10^-2^ |
| R00196 | reaction | (S)-Lactate:ferricytochrome-c 2-oxidoreductase | 1.00×10^-6^ |
| R00197 | reaction | (R)-Lactate:ferricytochrome-c 2-oxidoreductase | 1.82×10^-2^ |
| R00214 | reaction | (S)-malate:NAD+ oxidoreductase (decarboxylation) | 3.16×10^-2^ |
| R00226 | reaction | pyruvate:pyruvate acetaldehydetransferase (decarboxylation) | 1.43×10^-2^ |
| R00227 | reaction | Acetyl-CoA hydrolase | 1.00×10^-6^ |
| R00228 | reaction | acetaldehyde:NAD+ oxidoreductase (CoA-acetylation) | 7.48×10^-4^ |
| R00229 | reaction | acetate:CoA ligase (ADP-forming) | 1.00×10^-6^ |
| R00235 | reaction | Acetate:CoA ligase (AMP-forming) | 1.56×10^-4^ |
| R00315 | reaction | ATP:acetate phosphotransferase | 1.11×10^-3^ |
| R00317 | reaction | Acetyl phosphate phosphohydrolase | 2.19×10^-3^ |
| R00319 | reaction | (S)-lactate:oxygen 2-oxidoreductase (decarboxylation) | 1.00×10^-6^ |
| R00320 | reaction | diphosphate:acetate phosphotransferase | 1.00×10^-3^ |
| R00326 | reaction | acetaldehyde:acceptor oxidoreductase | 1.00×10^-6^ |
| R00605 | reaction | methanol:NAD+ oxidoreductase | 1.00×10^-6^ |
| R00608 | reaction | Methanol:oxygen oxidereductase | 1.00×10^-6^ |
| R00614 | reaction | formaldehyde:formaldehyde oxidoreductase | 1.00×10^-6^ |
| R00631 | reaction | aldehyde:NAD+ oxidoreductase | 1.00×10^-6^ |
| R00703 | reaction | (S)-Lactate:NAD+ oxidoreductase | 1.00×10^-6^ |
| R00710 | reaction | Acetaldehyde:NAD+ oxidoreductase | 1.00×10^-6^ |
| R00711 | reaction | Acetaldehyde:NADP+ oxidoreductase | 1.00×10^-6^ |
| R00743 | reaction | Acetyl-CoA:malonate CoA-transferase | 3.86×10^-2^ |
| R00746 | reaction | Ethanol:NADP+ oxidoreductase | 1.00×10^-6^ |
| R00749 | reaction | ethanolamine ammonia-lyase (acetaldehyde-forming) | 3.36×10^-2^ |
| R00753 | reaction | (S)-lactate acetaldehyde-lyase (formate-forming) | 1.00×10^-6^ |
| R00754 | reaction | ethanol:NAD+ oxidoreductase | 1.00×10^-6^ |
| R00897 | reaction | O3-acetyl-L-serine:hydrogen-sulfide 2-amino-2-carboxyethyltransferase | 3.40×10^-2^ |
| R00928 | reaction | Acetyl-CoA:propanoate CoA-transferase | 7.70×10^-5^ |
| R01026 | reaction | Acetylcholine aectylhydrolase | 2.56×10^-2^ |
| R01142 | reaction | Methane.NADH:oxygen oxidoreductase (hydroxylating) | 1.00×10^-6^ |
| R01146 | reaction | methanol:ferricytochrome-c oxidoreductase | 7.40×10^-6^ |
| R01179 | reaction | butanoyl-CoA:acetate CoA-transferase | 2.99×10^-2^ |
| R01200 | reaction | N-Acetyl-D-glucosamine amidohydrolase | 4.69×10^-2^ |
| R01315 | reaction | phosphatidylcholine 2-acylhydrolase | 1.80×10^-6^ |
| R01316 | reaction | phosphatidylcholine 1-acylhydrolase | 1.23×10^-5^ |
| R01348 | reaction | fatty acid.[reduced NADPH---hemoprotein reduction | 1.97×10^-2^ |
| R01350 | reaction | Glycerol acylhydrolase | 2.51×10^-5^ |
| R01351 | reaction | 1-acylglycerol acylhydrolase | 1.00×10^-6^ |
| R01359 | reaction | acetoacetyl-CoA:acetate CoA-transferase | 2.17×10^-4^ |
| R01366 | reaction | Acetoacetate carboxy-lyase (acetone-forming) | 1.00×10^-6^ |
| R01426 | reaction | Benzoate + Acetate + NADH + H+ <=> trans-Cinnamate + 2 H2O+ NAD+ | 1.49×10^-2^ |
| R01446 | reaction | (S)-lactaldehyde:NAD+ oxidoreductase | 1.00×10^-6^ |
| R01447 | reaction | (S)-Lactate:oxaloacetate oxidoreductase | 1.00×10^-6^ |
| R01449 | reaction | lactoyl-CoA:propanoate CoA-transferase | 1.00×10^-6^ |
| R01450 | reaction | lactate racemase | 1.00×10^-6^ |
| R01462 | reaction | cholesterol ester acylhydrolase | 1.23×10^-3^ |
| R01494 | reaction | N-Acylsphingosine amidohydrolase | 1.30×10^-6^ |
| R01550 | reaction | propan-2-ol:NADP+ oxidoreductase | 1.00×10^-6^ |
| R01553 | reaction | acetone-cyanohydrin acetone-lyase (cyanide-forming) | 1.00×10^-6^ |
| R02053 | reaction | phosphatidylethanolamine 2-acylhydrolase | 1.43×10^-3^ |
| R02054 | reaction | phosphatidylethanolamine 1-acylhydrolase | 1.27×10^-3^ |
| R02250 | reaction | triacylglycerol acylhydrolase | 2.13×10^-5^ |
| R02362 | reaction | Pectin pectylhydrolase | 1.00×10^-3^ |
| R02687 | reaction | 1.2-diacyl-sn-glycerol acylhydrolase | 1.00×10^-6^ |
| R02746 | reaction | 1-Acyl-sn-glycero-3-phosphocholine acylhydrolase | 2.86×10^-4^ |
| R02747 | reaction | 2-Acyl-sn-glycero-3-phosphocholine acylhydrolase | 5.54×10^-4^ |
| R02855 | reaction | (R)-Acetoin:NAD+ oxidoreductase | 1.00×10^-6^ |
| R02946 | reaction | (R.R)-Butane-2.3-diol:NAD+ oxidoreductase | 1.00×10^-6^ |
| R02948 | reaction | (S)-2-hydroxy-2-methyl-3-oxobutanoate carboxy-lyase | 1.00×10^-6^ |
| R02949 | reaction | (R)-Acetoin racemase | 1.00×10^-6^ |
| R03145 | reaction | pyruvate:ubiquinone oxidoreductase | 3.61×10^-4^ |
| R03416 | reaction | 1-Acyl-sn-glycero-3-phosphoethanolamine aldehydohydrolase | 3.82×10^-3^ |
| R03417 | reaction | L-2-Lysophosphatidylethanolamine aldehydohydrolase | 4.02×10^-3^ |
| R04471 | reaction | 1.2-Diacyl-3-beta-D-galactosyl-sn-glycerol acylhydrolase | 4.20×10^-2^ |
| R05198 | reaction | ethanol:cytochrome c oxidoreductase | 1.00×10^-6^ |
| R05917 | reaction | G00143 + H2O <=> G00144 + Acetate | 1.00×10^-6^ |
| R06518 | reaction | N-acylsphingosine amidohydrolase | 8.09×10^-4^ |
| R06528 | reaction | Phytoceramide amidohydrolase | 3.67×10^-2^ |
| R06973 | reaction | 3-oxopropanoate carboxy-lyase | 7.98×10^-3^ |
| R08388 | reaction | all-trans-retinyl ester acylhydrolase. 11-cis-retinol forming | 1.31×10^-2^ |
| R08974 | reaction | pheophorbide-a hydrolase (decarboxylating) | 3.55×10^-2^ |
| R09061 | reaction | chlorophyllide-a hydrolase | 5.26×10^-5^ |
| R09078 | reaction | (S)-acetoin:NAD+ oxidoreductase | 1.20×10^-3^ |
| R09098 | reaction | methanol:coenzyme M methyltransferase | 1.00×10^-6^ |
| R09127 | reaction | ethanol:cytochrome c oxidoreductase | 1.00×10^-6^ |
| R09479 | reaction | ethanol:quinone oxidoreductase | 1.00×10^-6^ |
| R09518 | reaction | methane.quinol:oxygen oxidoreductase | 1.00×10^-6^ |
| R09725 | reaction | pimeloyl-[acyl-carrier protein]-methyl-ester hydrolase | 2.59×10^-3^ |
| R09846 | reaction | prenylcycteine methylesterase | 3.65×10^-3^ |
| R10343 | reaction | succinyl-CoA:acetate CoA-transferase | 1.00×10^-6^ |
| R10504 | reaction | meso-2.3-butanediol:NAD+ oxidoreductase [(S)-acetoin forming] | 4.03×10^-3^ |
| R10505 | reaction | meso-2.3-butanediol:NAD+ oxidoreductase [(R)-acetoin forming] | 1.00×10^-6^ |
| R10506 | reaction | (S)-2-Acetolactate + Acceptor <=> Diacetyl + CO2 + Reduced acceptor | 1.06×10^-2^ |
| R10691 | reaction | 2.4-Diketo-3-deoxy-L-fuconate + H2O <=> (S)-Lactate + Pyruvate | 1.00×10^-6^ |
| R10703 | reaction | propan-2-ol:NAD+ oxidoreductase | 1.00×10^-6^ |
| R10704 | reaction | acetone.NADPH:oxygen oxidoreductase (methyl acetate-forming) | 1.00×10^-6^ |
| R10705 | reaction | methyl acetate acetohydrolase | 1.00×10^-6^ |
| R10912 | reaction | aldehyde:quinone oxidoreductase | 1.00×10^-6^ |
| R11074 | reaction | (S)-malate carboxy-lyase | 1.00×10^-6^ |
| C00033 | compound | Acetate | 1.00×10^-6^ |
| C00084 | compound | Acetaldehyde | 2.48×10^-5^ |
| C00132 | compound | Methanol | 1.00×10^-6^ |
| C00162 | compound | Fatty acid | 1.00×10^-6^ |
| C00164 | compound | Acetoacetate | 4.77×10^-2^ |
| C00186 | compound | Lactate | 1.00×10^-6^ |
| C00207 | compound | Acetone | 1.00×10^-6^ |
| C00469 | compound | Ethanol | 1.00×10^-6^ |
| C00810 | compound | Acetoin | 1.00×10^-6^ |
| C01326 | compound | Hydrogen cyanide | 3.23×10^-2^ |
| C01769 | compound | (S)-Acetoin | 1.28×10^-3^ |
| C01845 | compound | Propan-2-ol | 1.61×10^-2^ |
| C02659 | compound | Acetone cyanohydrin | 3.92×10^-2^ |
| C06010 | compound | 2-Acetolactate | 2.17×10^-2^ |

**Table S3.** Gene-metabolite network analysis identifying the genes perturbed in post-COVID-19 patients. Genes are grouped according to different degrees of interaction with metabolites.

| ***Label*** | ***Degree*** | ***Betweenness*** | ***KEGG name*** |  |
| --- | --- | --- | --- | --- |
| Ethanol | 375 | 128170.35 |  | **Metabolites** |
| Methanol | 85 | 31774.98 |  |  |
| l-Lactic acid | 32 | 12290.35 |  |  |
| Isovaleric acid | 28 | 12183 |  |  |
| Acetone | 24 | 6854.97 |  |  |
| Acetoin | 20 | 7092.35 |  |  |
| DECR1 | 3 | 3267.73 | 2.4-Dienoyl-CoA reductase 1 | **Genes with 3 degrees of interaction with metabolites** |
| ABHD6 | 3 | 2265.7 | abHydrolase domain containing 6. acylglycerol lipase |  |
| CYP2E1 | 3 | 2265.7 | Cytochrome P450 family 2 subfamily E member 1 |  |
| CYB5A | 3 | 2265.7 | Cytochrome b5 type A |  |
| CTSD | 2 | 3075 | Cathepsin D | **Genes with 2 degrees of interaction with metabolites** |
| REN | 2 | 3075 | Renin |  |
| PLA2G1B | 2 | 3075 | Phospholipase A2 group IB |  |
| PGA3 | 2 | 3075 | Pepsinogen A3 |  |
| LAT2 | 2 | 2202.35 | Linker for activation of T cells family member 2 |  |
| GPD1 | 2 | 2202.35 | Glycerol-3-phosphate dehydrogenase 1 |  |
| FOS | 2 | 1983.99 | Fos proto-oncogene. AP-1 transcription factor subunit |  |
| PKLR | 2 | 1983.99 | Pyruvate kinase L/R |  |
| GCG | 2 | 1983.99 | Glucagon |  |
| PKM2 | 2 | 1983.99 | Pyruvate kinase M1/2 |  |
| PC | 2 | 1983.99 | Pyruvate carboxylase |  |
| BGLAP | 2 | 1983.99 | Bone gamma-carboxyglutamate protein |  |
| CAT | 2 | 1454.45 | Catalase |  |
| SSB | 2 | 1454.45 | small RNA binding exonuclease protection factor La |  |
| CYP1A2 | 2 | 1454.45 | Cytochrome P450 family 1 subfamily A member 2 |  |
| ALPI | 2 | 1454.45 | Alkaline phosphatase. intestinal |  |
| ALB | 2 | 1454.45 | Albumin |  |
| ACHE | 2 | 1454.45 | Acetylcholinesterase |  |
| ENTPD1 | 2 | 1454.45 | Ectonucleoside triphosphate diphosphohydrolase 1 |  |
| ADH7 | 2 | 1454.45 | Alcohol dehydrogenase 7 |  |
| ADH1A | 2 | 1454.45 | Alcohol dehydrogenase 1A | **Genes with 2 degrees of interaction with metabolites** |
| ADH4 | 2 | 1454.45 | Alcohol dehydrogenase 4 |  |
| PTGR2 | 2 | 1454.45 | Prostaglandin reductase 2 |  |
| ADH1B | 2 | 1454.45 | Alcohol dehydrogenase 1B |  |
| ADH6 | 2 | 1454.45 | Alcohol dehydrogenase 6 |  |
| ADHFE1 | 2 | 1454.45 | Alcohol dehydrogenase iron containing 1 |  |
| ADH5 | 2 | 1454.45 | Alcohol dehydrogenase 5 |  |
| AVP | 2 | 1454.45 | Arginine vasopressin |  |
| CYP3A5 | 2 | 628.34 | Cytochrome P450 family 3 subfamily A member 5 |  |
| MBD2 | 2 | 628.34 | Methyl-CpG binding domain protein 2 |  |
| LPL | 2 | 628.34 | Lipoprotein lipase |  |
| ODC1 | 2 | 628.34 | Ornithine decarboxylase 1 |  |
| COQ6 | 2 | 628.34 | Coenzyme Q6. monooxygenase |  |
| TARBP2 | 2 | 182.91 | TARBP2 subunit of RISC loading complex |  |
| CSTB | 2 | 182.91 | Cystatin B |  |
| PTCHD3 | 2 | 182.91 | Patched domain containing 3 |  |
| LDHA | 2 | 78.18 | Lactate dehydrogenase A |  |
| LDHB | 2 | 78.18 | Lactate dehydrogenase B |  |
| LDHC | 2 | 78.18 | Lactate dehydrogenase C |  |
| LDHAL6A | 2 | 78.18 | Lactate dehydrogenase A like 6A |  |
| LDHAL6B | 2 | 78.18 | Lactate dehydrogenase A like 6B |  |

**Table S4**. Functional enrichment miRNet analysis resulted in 6 significant miRNA-Functions with more than 60 Hits.

| **miRNA-Function name** | **Gene Ontology annotation** | **Hits** | ***p* value** |
| --- | --- | --- | --- |
| Cell cycle | GO:0007049 | 74 | 0.00289 |
| Regulation of stem cell proliferation | GO:0072091 | 74 | 0.00707 |
| Cell death | GO:0008219 | 73 | 0.0153 |
| Aging | GO:0007568 | 70 | 0.034 |
| Hematopoiesis | GO:0030097 | 68 | 0.00486 |
| Angiogenesis | GO:0001525 | 66 | 0.0365 |


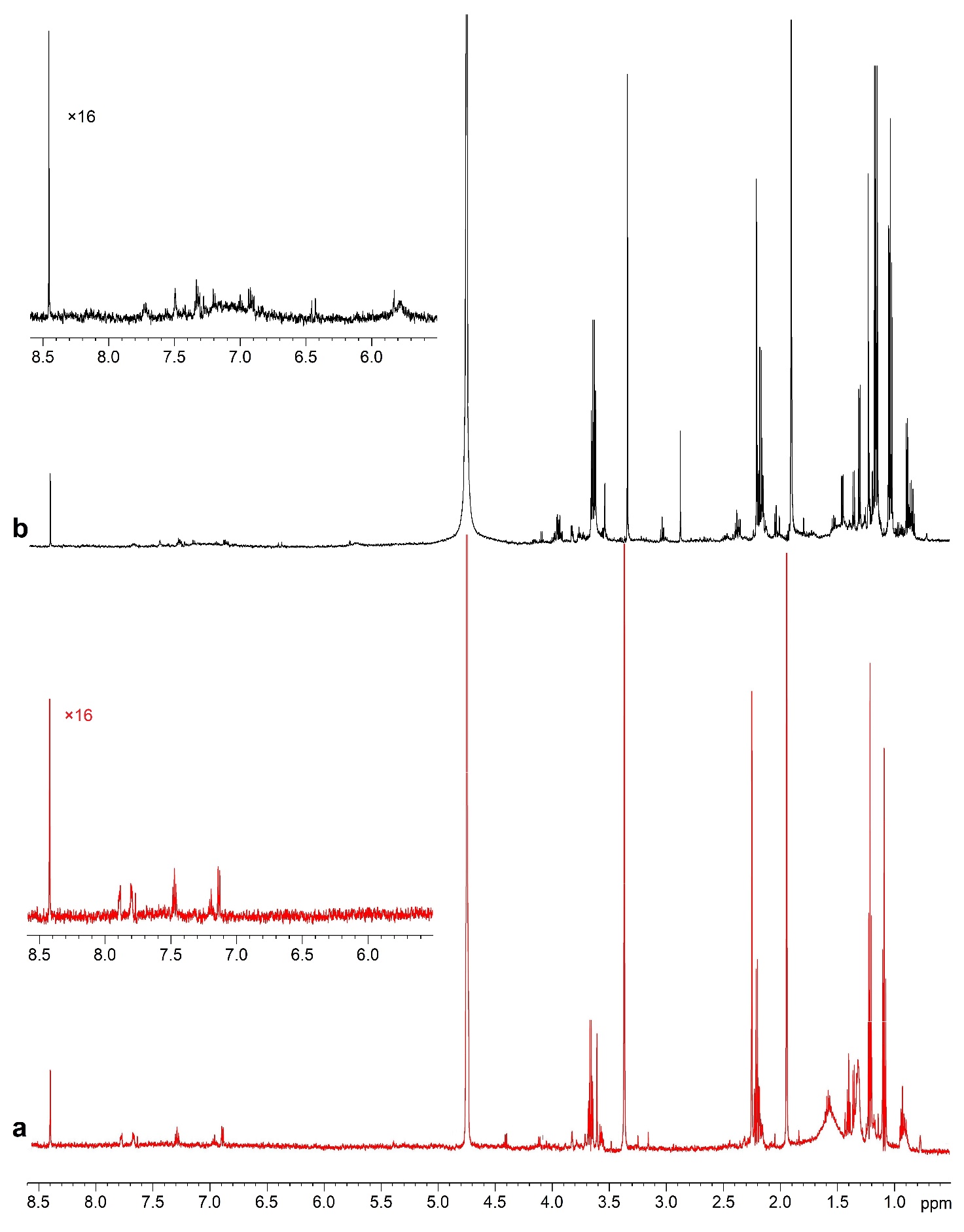


**Fig. S1.** NMR spectrum of EBC samples. Comparison between representative one-dimensional ^1^H-NMR spectra of an EBC sample from a post-COVID patient (**b**, black trace) and a healthy subject (**a**, red trace). The 8.5−5.5 ppm regions have been vertically expanded 16 times with respect to the full spectrum. The resonances’ identification is reported in Table S1.


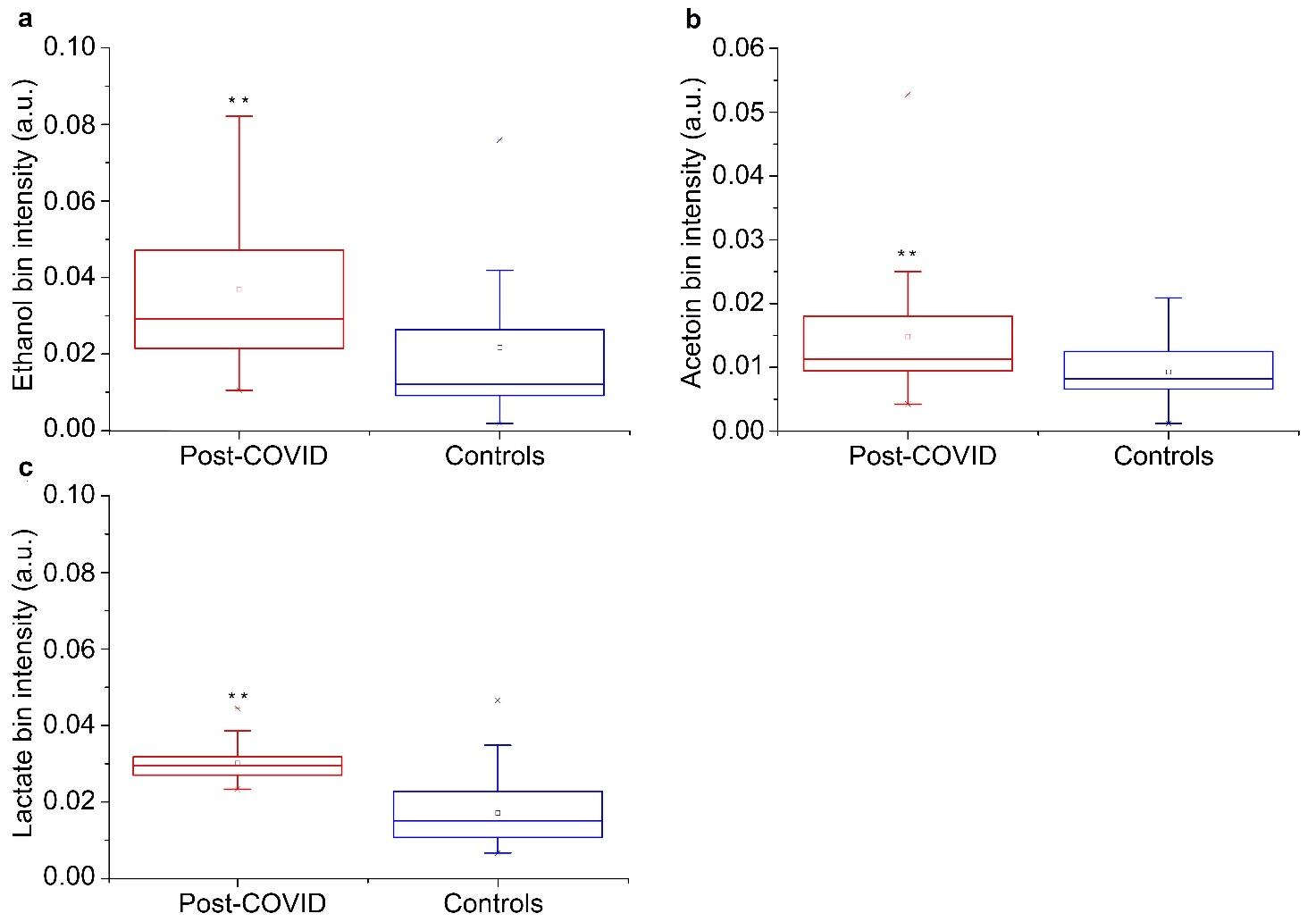


**Fig. S2.** Box-and-whisker plots showing the concentration levels of the metabolites discriminating post-COVID-19 patients and controls. (**a**) Ethanol; (**b**) acetoin; (**c**) lactate. Boxes show median (horizontal line in each box). the mean (the empty box). 25th and 75th percentiles (edges of box). maximum and minimum values (whiskers). and the outliers (cross). ANOVA test significance is reported as **. *p*< 0.001.


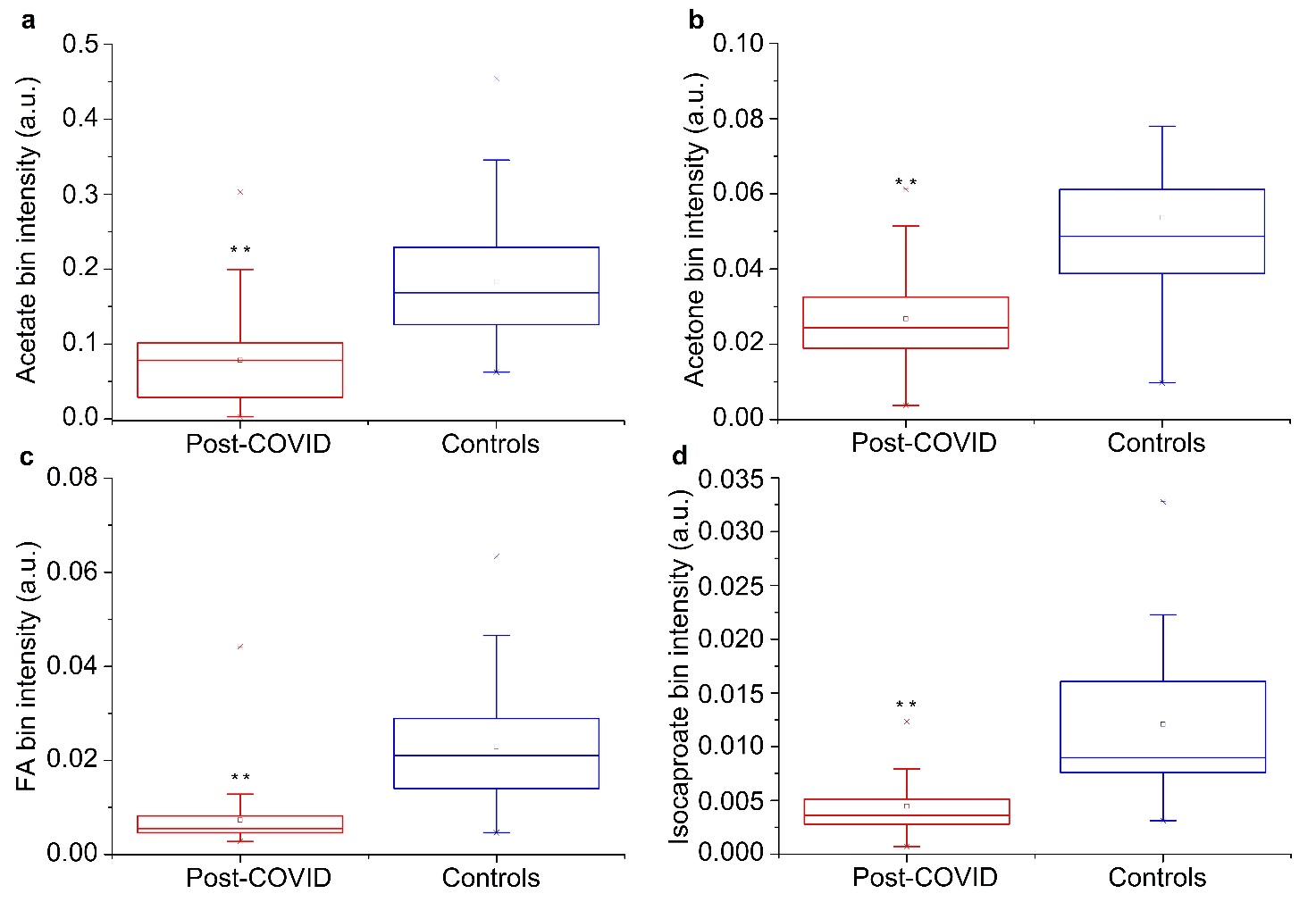


**Fig. S3.** Box-and-whisker plots showing the concentration levels of the metabolites discriminating post-COVID-19 patients and controls. (**a**) Acetate; (**b**) acetone; (**c**) fatty acids (FA); (**d**) isocaproate. Boxes show median (horizontal line in each box). the mean (the empty box). 25th and 75th percentiles (edges of box). maximum and minimum values (whiskers). and the outliers (cross). ANOVA test significance is reported as **. *p*< 0.001.


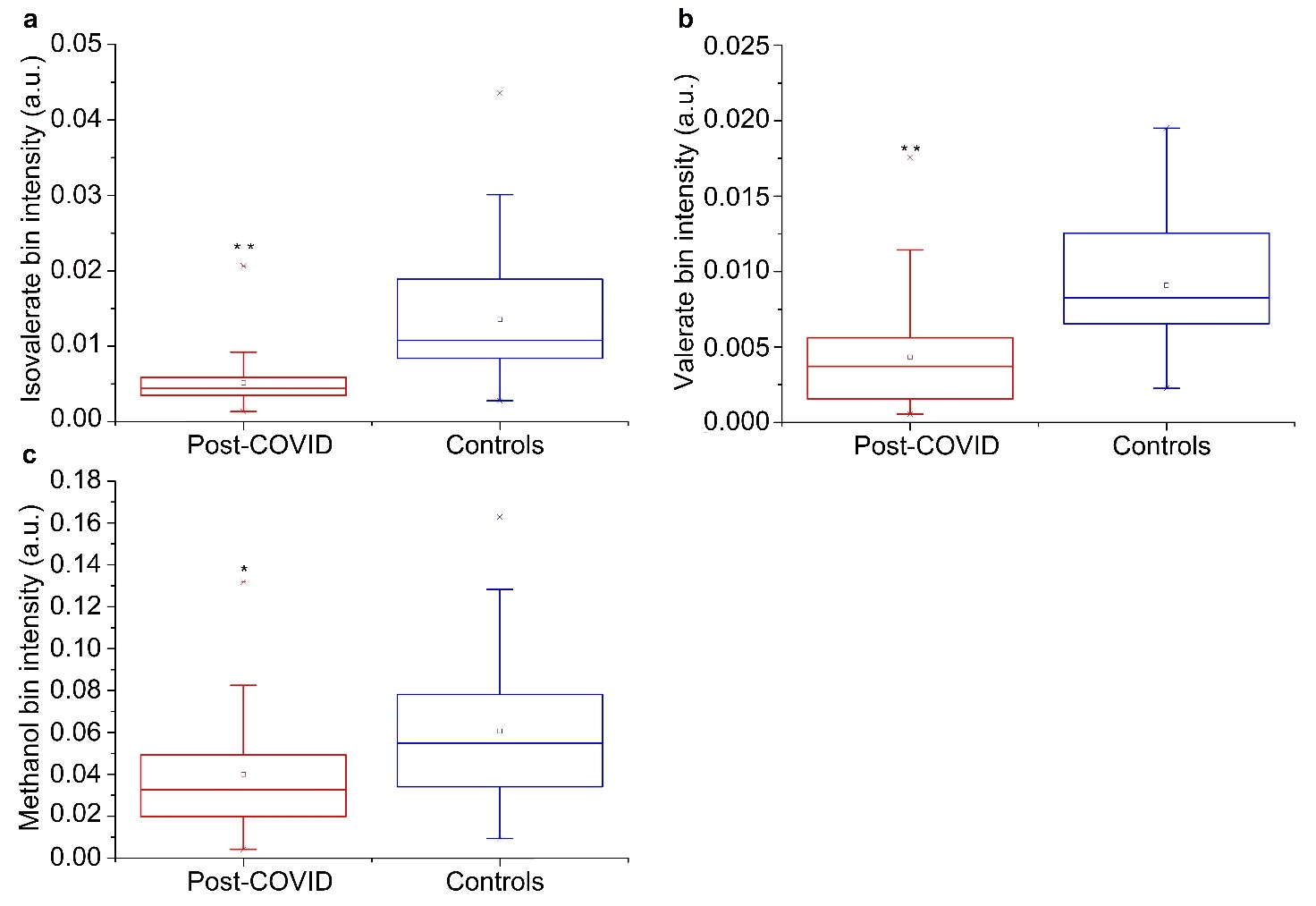


**Fig. S4.** Box-and-whisker plots showing the concentration levels of the metabolites discriminating post-COVID-19 patients and controls. (**a**) isovalerate; (**b**) valerate; (**c**) methanol. Boxes show median (horizontal line in each box). the mean (the empty box). 25th and 75th percentiles (edges of box). maximum and minimum values (whiskers). and the outliers (cross). ANOVA test significance is reported as *. *p*< 0.05 and **. *p*< 0.001.
